# Multiple roles of the non-structural protein 3 (nsP3) alphavirus unique domain (AUD) during Chikungunya virus genome replication and transcription

**DOI:** 10.1371/journal.ppat.1007239

**Published:** 2019-01-22

**Authors:** Yanni Gao, Niluka Goonawardane, Joseph Ward, Andrew Tuplin, Mark Harris

**Affiliations:** School of Molecular and Cellular Biology, Faculty of Biological Sciences and Astbury Centre for Structural Molecular Biology, University of Leeds, Leeds, United Kingdom; Purdue University, UNITED STATES

## Abstract

Chikungunya virus (CHIKV) is a re-emerging *Alphavirus* causing fever, joint pain, skin rash, arthralgia, and occasionally death. Antiviral therapies and/or effective vaccines are urgently required. CHIKV biology is poorly understood, in particular the functions of the non-structural protein 3 (nsP3). Here we present the results of a mutagenic analysis of the alphavirus unique domain (AUD) of nsP3. Informed by the structure of the Sindbis virus AUD and an alignment of amino acid sequences of multiple alphaviruses, a series of mutations in the AUD were generated in a CHIKV sub-genomic replicon. This analysis revealed an essential role for the AUD in CHIKV RNA replication, with mutants exhibiting species- and cell-type specific phenotypes. To test if the AUD played a role in other stages of the virus lifecycle, the mutants were analysed in the context of infectious CHIKV. This analysis indicated that the AUD was also required for virus assembly. In particular, one mutant (P247A/V248A) exhibited a dramatic reduction in production of infectious virus. This phenotype was shown to be due to a block in transcription of the subgenomic RNA leading to reduced synthesis of the structural proteins and a concomitant reduction in virus production. This phenotype could be further explained by both a reduction in the binding of the P247A/V248A mutant nsP3 to viral genomic RNA *in vivo*, and the reduced affinity of the mutant AUD for the subgenomic promoter RNA *in vitro*. We propose that the AUD is a pleiotropic protein domain, with multiple functions during CHIKV RNA synthesis.

## Author summary

Chikungunya virus (CHIKV) is an emerging threat to world health. It is transmitted by Aedes species mosquitos, and has caused massive epidemics across the globe. The virus causes fever, rash, arthritis and can sometimes be fatal. The biology of CHIKV is poorly understood, to address this deficiency we aimed to identify functions of one of the viral proteins, nsP3. We focused on the central part of this protein, termed the alphavirus unique domain (AUD) because it is unique to the genus of viruses to which CHIKV belongs–the *Alphaviruses*–and not present in other related viruses. By making changes (mutations) in the amino acid sequence of the AUD and analysing the effects of these changes we show that it is involved in multiple stages of the virus lifecycle. These observations identify nsP3 and the AUD in particular as a potential target for antiviral therapy or rational vaccine design.

## Introduction

Chikungunya virus (CHIKV; family *Togaviridae*, genus *Alphavirus*) [1] is an arbovirus that causes fever, rash and arthralgia with an infrequent fatal outcome [2]. It was first isolated in Tanzania in 1952–1953 [3,4]. During the last 50 years, numerous CHIKV re-emergences have been documented across the world, including Africa, Asia, Europe and America [5,6]. CHIKV is transmitted to humans by mosquitoes, mainly *Aedes aegypti* and *Aedes albopictus*. The latter can reproduce in more moderate climates meaning that CHIKV has spread from Southern Africa and is now present across the Americas and parts of Southern Europe (including France and Italy). Increasing global temperatures resulting from climate change raise the concern that CHIKV will spread further. In this regard, there are no antiviral therapies or safe, effective vaccines available to treat CHIKV infection.

**Fig 1:**
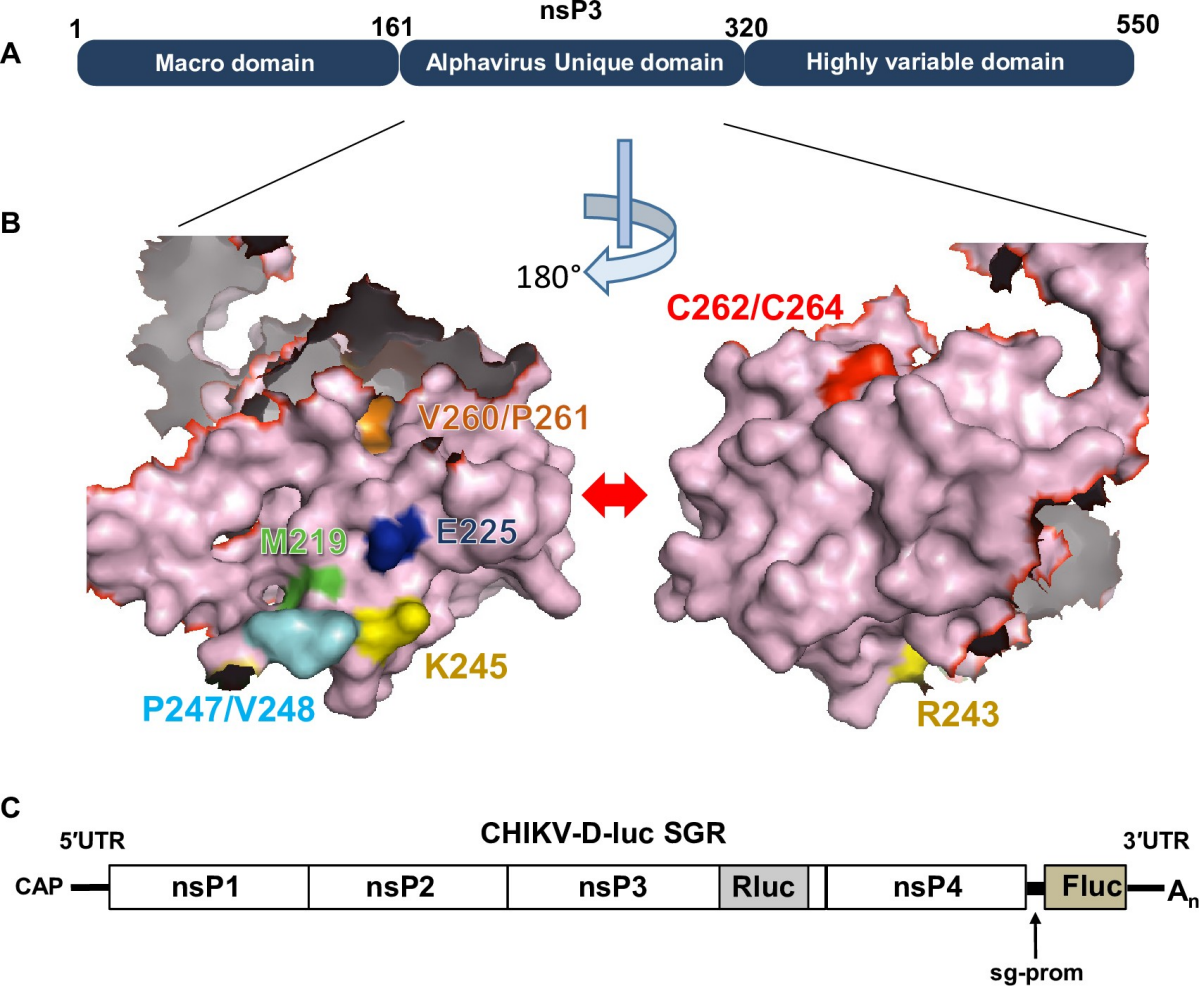
(A) Schematic of the domain structure of the alphavirus nsP3 protein. (B) Surface representation of the Sindbis virus nsP3 AUD structure (PDB ID code 4GUA) [14] (residues 161–320), including the 40 amino acid flexible linker between the macrodomain and the AUD. The locations of the mutated residues in nsP3 are highlighted in colour with their designation alongside in text of the same colour. The two images show opposite faces of the structure, rotated 180^o^ along the vertical axis. (C) Structure of CHIKV-D-Luc-SGR. This replicon was derived from the CHIKV ECSA strain LR2006 OPY1 isolate [15,16] and is described in [17]. Renilla luciferase (RLuc) is expressed as an internal fusion with nsP3 and thus is produced following translation of the input RNA. RLuc activity is therefore an indirect measure of both input translation and subsequent genome replication. Firefly luciferase (FLuc) is expressed from the subgenomic promoter (sg-prom) and thus is only produced after genome replication has occurred (synthesis of negative strand RNA that subsequently templates the production of both full-length genomic and sgRNA).

CHIKV has an 11.5 kilobase positive-sense, single-stranded RNA genome that is both capped and polyadenylated, and contains two open reading frames (ORFs). The first ORF is translated directly from full-length genomic RNA and encodes the non-structural proteins nsP1 to nsP4. These four proteins are required for RNA synthesis–generating both negative and positive full-length genomic RNA and a smaller subgenomic RNA from which the second ORF is translated to yield the structural proteins (capsid, envelope glycoproteins E1-3 and the 6K viroporin). Biochemical functions have been ascribed to 3 of the nsPs: nsP1 exhibits methyl- and guanyl-transferase activities, nsP2 is a helicase/protease, and nsP4 is the RNA-dependent RNA polymerase. Although nsP3 plays an essential role in RNA replication, its biochemical functions remain largely undefined [7,8]. It is proposed to comprise three domains: at the N-terminus is a macro-domain which exhibits both ADP-ribose and RNA binding, and ADP-ribosylhydrolase capabilities [9–11]. This is followed by the alphavirus unique domain (AUD), so called as it is only present in the *Alphavirus* genus and is absent from the closely related Rubella virus (the sole member of the *Rubivirus* genus within the *Togaviridae*), and a C-terminal hypervariable region (Fig 1A). The latter plays an important role in virus-host interactions and may be a significant determinant of pathogenesis through interactions with cell-type specific factors [12,13]. The AUD is located in the centre of nsP3, and despite a high level of sequence homology across the alphaviruses, the function of this domain remains elusive. Of note, the structure of the Sindbis virus (SINV) AUD has been determined in the context of a pre-cleavage fragment of the polyprotein spanning the C-terminus of nsP2 (protease and methyl-transferase-like domains), and the N-terminus of nsP3 (macro-domain and AUD) [14]. This revealed that the AUD presents a unique protein fold containing a zinc coordination site. In this study we sought to investigate the function of AUD during the virus lifecycle in cells derived from both the vertebrate host and the mosquito vector, to identify targets for antiviral intervention and means of rational attenuation for vaccine development. By mutagenic analysis we demonstrate that the AUD exhibits both species- and cell-type specific phenotypes, and plays roles in both virus genome replication and structural protein expression.

## Results

### Construction of CHIKV subgenomic replicons with AUD mutations

To identify residues within the AUD that are conserved across the *Alphavirus* genus we first aligned the AUD amino acid sequences of a range of both Old World and New World alphaviruses (S1A Fig). As the AUD sequences between SINV and CHIKV are highly conserved (118 of 243 residues are identical), the nsP2/nsP3 protein structure of SINV [14] was referenced to identify the putative location of each of the conserved residues. Following from the above analysis, 10 residues were chosen for further study as they were located on the surface of the protein (S1B Fig) and were either absolutely conserved throughout the alphaviruses, or in other cases were substituted by residues with similar physical characteristics (specifically the corresponding residue for both Met219 and Val260 in CHIKV is leucine in SINV) (Fig 1B and S1A Fig). We chose to make two single substitutions (nsP3 amino acid numbering: M219A and E225A), and four double substitutions of adjacent or closely located residues (R243A/K245A, P247A/V248A, V260A/P261A and C262A/C264A). The latter two residues were shown to be involved in zinc coordination in the SINV AUD domain [14]. These mutations were introduced into a CHIKV subgenomic replicon (CHIKV-D-Luc-SGR) (Fig 1C), derived from the ECSA strain LR2006 OPY1 isolate [15,16]. This construct contains two luciferase reporter genes, a renilla luciferase (RLuc) is fused in frame within the C-terminal hypervariable domain of nsP3 in ORF1, and a firefly luciferase (FLuc) replaces the structural protein encoding region of ORF2 [17]. RLuc activity is thus a measure of translation from the full-length RNA and at early time points allows assessment of both transfection efficiency and input translation. The subgenomic promoter (sg-prom) drives production of the sgRNA which requires synthesis of negative strand RNA, FLuc expression thus allows assessment of RNA replication. As a negative control we also created a polymerase-inactive mutant (GDD-GAA in the active site of nsP4).

### CHIKV subgenomic replicons exhibited different phenotypes in human, mammalian and mosquito cells

To analyse the effects of the AUD mutations on CHIKV genome replication, the panel of mutant CHIKV-D-Luc-SGR RNAs were transfected into a range of cell lines. As both liver and muscle are target organs for CHIKV infection we used three human cell lines. The human hepatoma cell line Huh7 is well characterised and we have previously shown that they efficiently support CHIKV replication [17]. To test any potential role of the AUD in protecting CHIKV from innate immune sensing we also used the Huh7 derivative Huh7.5, which have a defect in innate immunity due in part to a mutation in one allele of the retinoic acid-inducible gene I (RIG-I) [18]. Indeed, it was shown that ectopic expression of wildtype RIG-I in Huh7.5 restored the activation of IRF-3 and expression of ISGs in response to Sendai virus [18]. To investigate the role of the AUD during CHIKV genome replication in muscle cells we used a human rhabdomyosarcoma cell line, RD. Additionally, two other mammalian (non-human) cell lines were used: C2C12 (a murine myoblast cell line) and BHK-21 (baby hamster kidney cells) based on their ability to support high levels of CHIKV replication [17]. Lastly, we used two mosquito (*Aedes albopictus*) derived cell lines: U4.4 and C6/36. Of note, C6/36 have a defect in RNA interference (RNAi) due to a frameshift mutation in the Dcr2 gene, leading to production of a truncated and inactive Dicer-2 protein [19]. Again, use of these cells was intended to allow us to assess any role of the AUD in counteracting mosquito innate immunity. To allow direct comparison of replication between different cell lines, all luciferase values are presented as fold increase over the 4 h values, absolute luciferase values are presented in S2 Fig (FLuc) and S3 Fig (RLuc).

**Fig 2:**
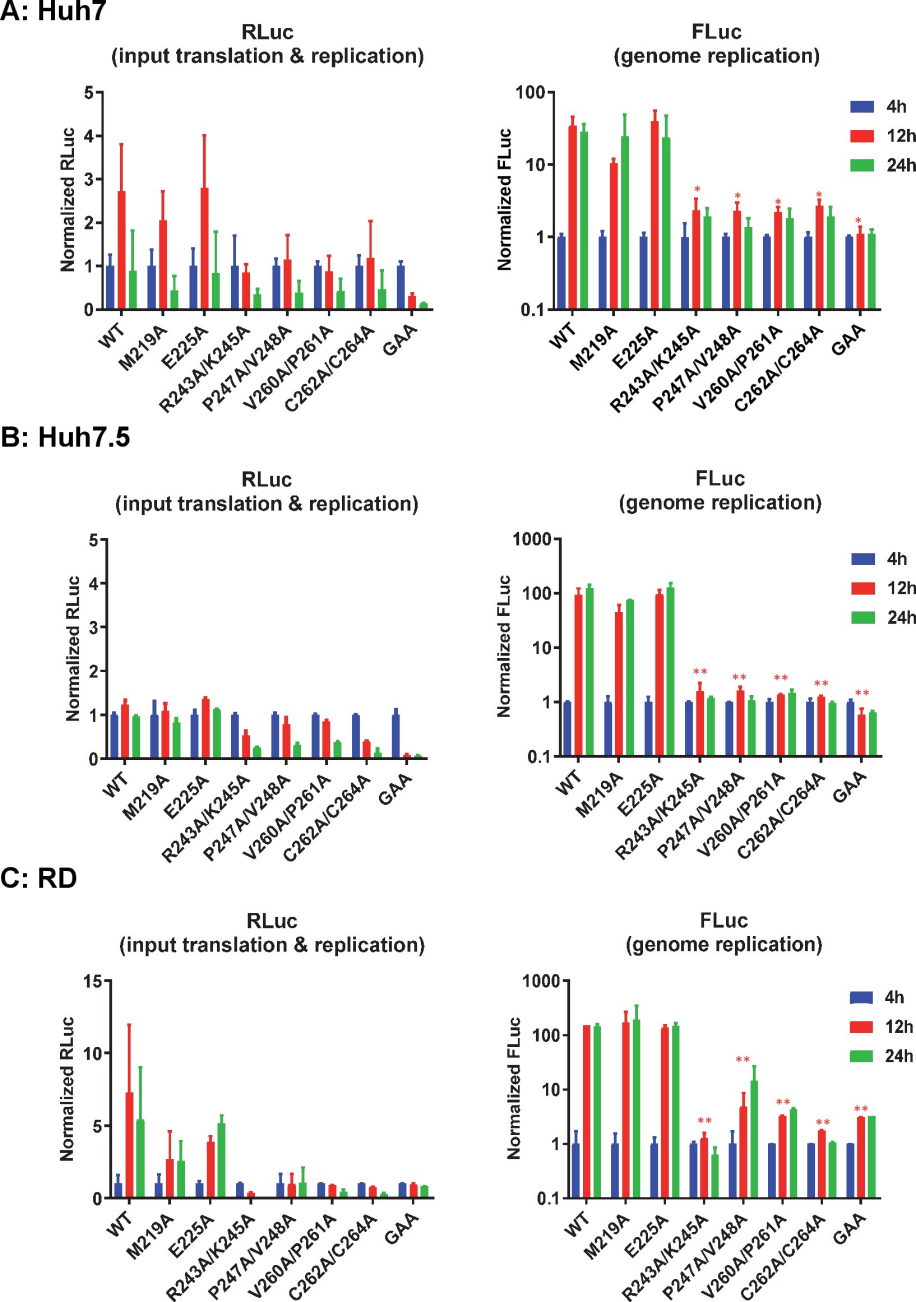
CHIKV AUD mutant replication in human cells. Huh7 (A), Huh7.5 (B) or RD cells (C) were transfected with CHIKV-D-luc-SGR wildtype (WT) and mutant RNAs and harvested for both RLuc and FLuc assays at the indicated time points. Luciferase values of wildtype and each mutant were normalized to 4 h values and represent fold-change compared to the 4 h values. (GAA: inactive mutant of nsP4 polymerase in a wildtype nsP3 background). Significant differences denoted by * (P<0.05), and ** (P<0.01), compared to wildtype are only shown for the 12 h timepoint values for clarity. Data are displayed as the means ± S.E. of three experimental replicates.

We first tested replication in the human hepatoma cell line, Huh7 (Fig 2A). Wildtype CHIKV-D-Luc-SGR exhibited robust replication in these cells with FLuc levels (a measure of genome replication) increasing approx. 30-fold between 4–12 h post-transfection (h.p.t.). Consistent with this, RLuc levels (reflecting both input translation and subsequent replication) increased between 4–12 h.p.t., but then declined at 24 h.p.t. to input levels, possibly due to preferential transcription of the sub-genomic RNA at later times. Of the mutants M219A exhibited a modest, non-significant, reduction in replication, E225A replicated as wild type, but the other four nsP3 mutants and the nsP4 GAA mutant failed to replicate. The replication defect was indicated by either a reduction or a minimal increase in both RLuc and FLuc values from 4–24 h.p.t. A similar picture emerged when the mutant panel was screened in Huh7.5 cells (Fig 2B). Consistent with the defect in cytosolic RNA sensing, replication of wildtype, M219A and E225A was higher in Huh7.5 cells compared to Huh7, however, this did not allow replication of the inactive mutants. Intriguingly, this enhanced replication in Huh7.5 cells did not manifest in higher levels of RLuc expression at 12 h.p.t. compared to 4 h.p.t. (as compared to Huh7 cells), this may not be due to effects on replication but more likely reflect a preference for translation of the sgRNA. For RD human rhabdomyosarcoma cells, a slightly different picture emerged (Fig 2C): firstly both M219A and E225A replicated to a similar level as wildtype. Secondly, the P247A/V248A mutant, which was unable to replicate in Huh7 or Huh7.5 cells, was able to replicate to a low level in RD cells. The other three mutants and nsP4 GAA again failed to replicate.

**Fig 3:**
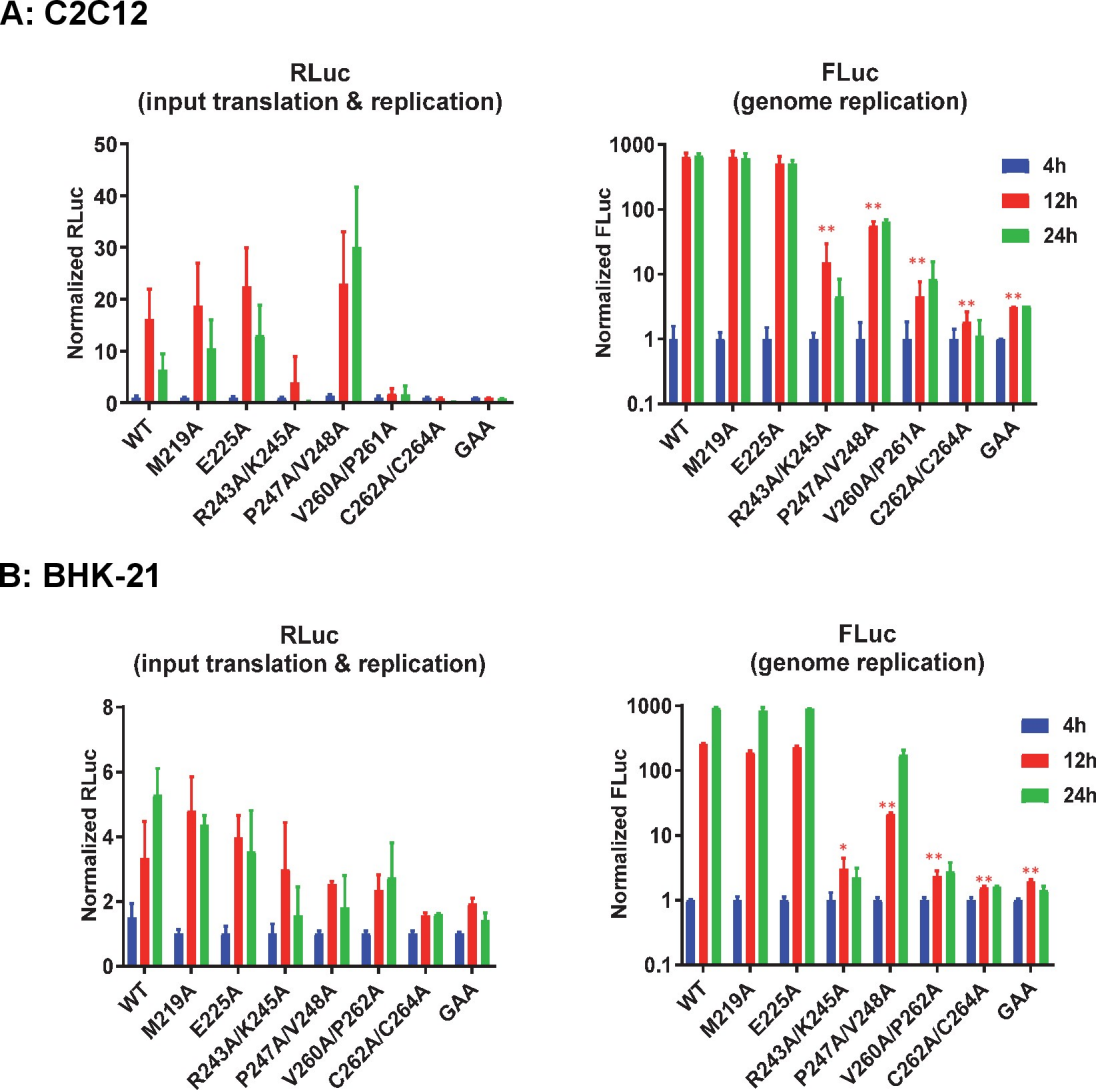
CHIKV AUD mutant replication in murine cells. C2C12 (A) or BHK-21 cells (B) were transfected with CHIKV-D-luc-SGR wildtype (WT) and mutant RNAs and harvested for both RLuc and FLuc assays at the indicated time points. Luciferase values of wildtype and each mutant were normalized to 4 h values and represent fold-change compared to the 4 h values. (GAA: inactive mutant of nsP4 polymerase in a wildtype nsP3 background). Significant differences denoted by * (P<0.05), and ** (P<0.01), compared to wildtype are only shown for the 12 h timepoint values for clarity. Data are displayed as the means ± S.E. of three experimental replicates.

We then evaluated the mutant panel in two other mammalian cell lines: C2C12 murine myoblasts (Fig 3A) and BHK-21 (Fig 3B). Wildtype CHIKV-D-Luc-SGR replicated to very high levels in both cell lines, much higher than in the human cell lines (Fig 2), with FLuc levels increasing ~1000-fold between 4–24 h.p.t. Overall the phenotypes of the panel of mutants were similar to those observed in RD, however two noticeable differences were observed. Firstly, R243A/K245A showed a low replication level in C2C12 cells. Secondly, P247A/V248A was capable of replication in both but at levels that were significantly lower than wildtype (>10-fold). Interestingly, although in C2C12 cells FLuc levels for P247A/V248A were reduced, the concomitant RLuc levels were higher than wildtype, suggesting that there may be a defect in either transcription of the sgRNA or translation of ORF2. These data suggested that P247 and V248 were required for CHIKV genome replication in liver-derived cells, whilst enhancing but not essential for replication in cells derived from muscle or kidney, implying some cell-type specific interactions of nsP3. V260A/P261A (adjacent to the zinc–binding site), and the zinc-coordinating cysteine mutant C262A/C264A were unable to replicate in either cell line, being indistinguishable from the GAA nsP4 control.

**Fig 4:**
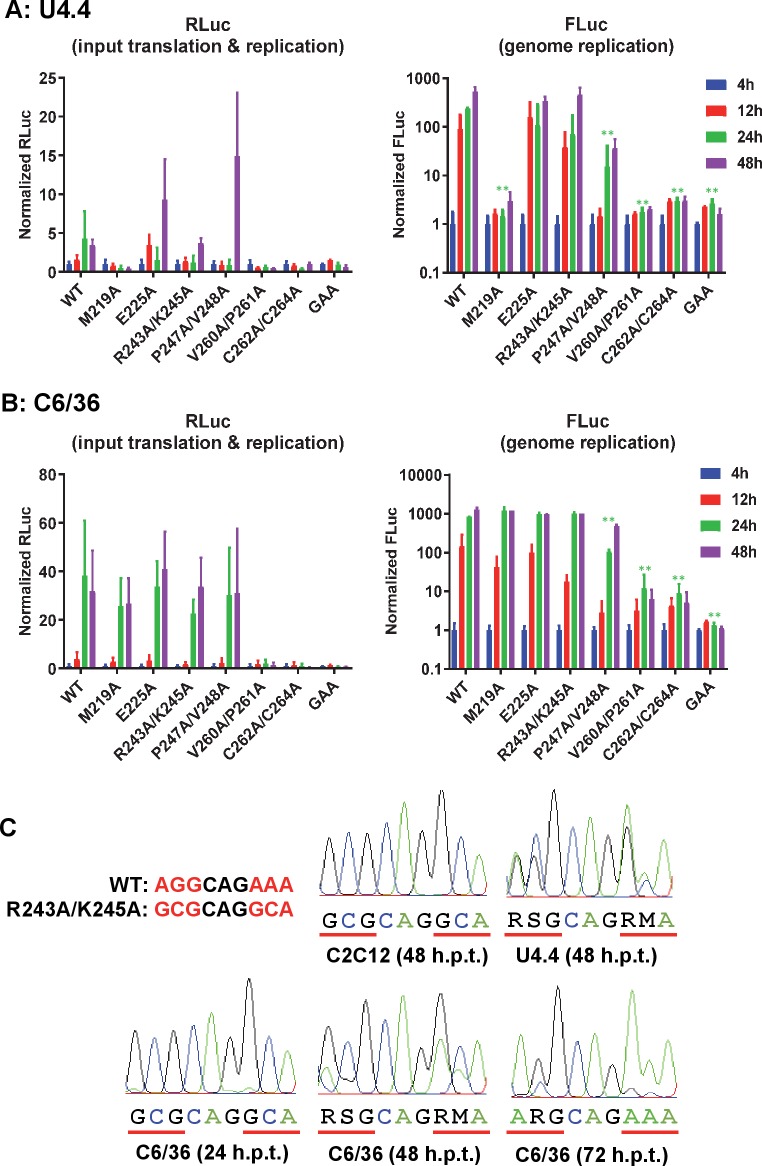
CHIKV AUD mutant replication in *Aedes albopictus* mosquito cells. U4.4 (A) or C6/36 cells (B) were transfected with CHIKV-D-luc-SGR wildtype (WT) and mutant RNAs and harvested for both renilla and firefly luciferase assay at the indicated time points. Luciferase values of wildtype and each mutant were normalized to 4 h values and represent fold-change compared to the 4 h values. (GAA: inactive mutant of nsP4 polymerase in a wildtype nsP3 background). Significant differences denoted by ** (P<0.01), compared to wildtype are only shown for the 24 h timepoint values for clarity. Data are displayed as the means ± S.E. of three experimental replicates. (C) RT-PCR and sequencing analysis of CHIKV-D-luc-SGR-R243A/K245A. RNA was harvested at the indicated times, amplified by RT-PCR and sequenced. The wildtype and R243A/K245A mutant sequences are shown alongside the sequence traces for reference. Nucleotide ambiguity codes used: R (A/G), S (G/C) and M (A/C).

As a mosquito transmitted virus, CHIKV must replicate in both mammalian and mosquito cells. We therefore proceeded to evaluate the replicative capacity of the mutant panel in cells derived from the *Aedes albopictus* mosquito. Two cell lines were used: U4.4 and C6/36. The major difference between these two cell lines is that C6/36 have a defect in the RNAi response due to a frameshift mutation in the Dcr2 gene [19]. Consistent with this, although both mosquito cell lines supported robust replication, C6/36 supported higher levels than U4.4 (up to 1000-fold increase at 48 h.p.t.). As described below, we observed remarkable differences in the mutant phenotypes in these cells compared to the mammalian cells (Fig 4). The first difference was that M219A failed to replicate in U4.4 cells (Fig 4A) but exhibited wildtype levels of replication in C6/36 cells (Fig 4B), suggesting that M219 might be involved in interacting with, and inhibiting, the mosquito cell RNAi pathway. Secondly, R243A/K245A, which was unable to replicate in human cell lines and only showed low replication level in the C2C12 cells, was fully replication competent in both mosquito cell lines. Mutant P247A/V248A was partially replication competent in both cell lines, whereas as seen in mammalian cell lines neither V260A/P261A nor C262A/C264A replicated in mosquito cells.

The striking phenotypic difference between mammalian and mosquito cell lines for R243A/K245A led us to investigate this further. We considered that a simple explanation might be that the mutations had reverted to wildtype in mosquito cell lines. To test this we extracted cytoplasmic RNA from transfected C2C12, U4.4 and C6/36 cells, and subjected them to RT-PCR and sequence analysis. In C2C12 cells at 48 h.p.t. we did not observe any sign of reversion (Fig 4C)–the sequence remained the same as the input RNA. However, for both U4.4 at 48 h.p.t., and C6/36 samples the sequence traces revealed the presence of a mixed population of mutant and wildtype. Notably we observed a sequential accumulation of revertants in the C6/36 samples: At 24 h.p.t. a very low proportion of revertants at the first position in the two codons was seen, at 48 h.p.t. the proportion increased and at 72 h.p.t. the sequences were almost entirely wildtype. These data are consistent with a requirement for R243 and K245 for efficient CHIKV genome replication.

**Fig 5:**
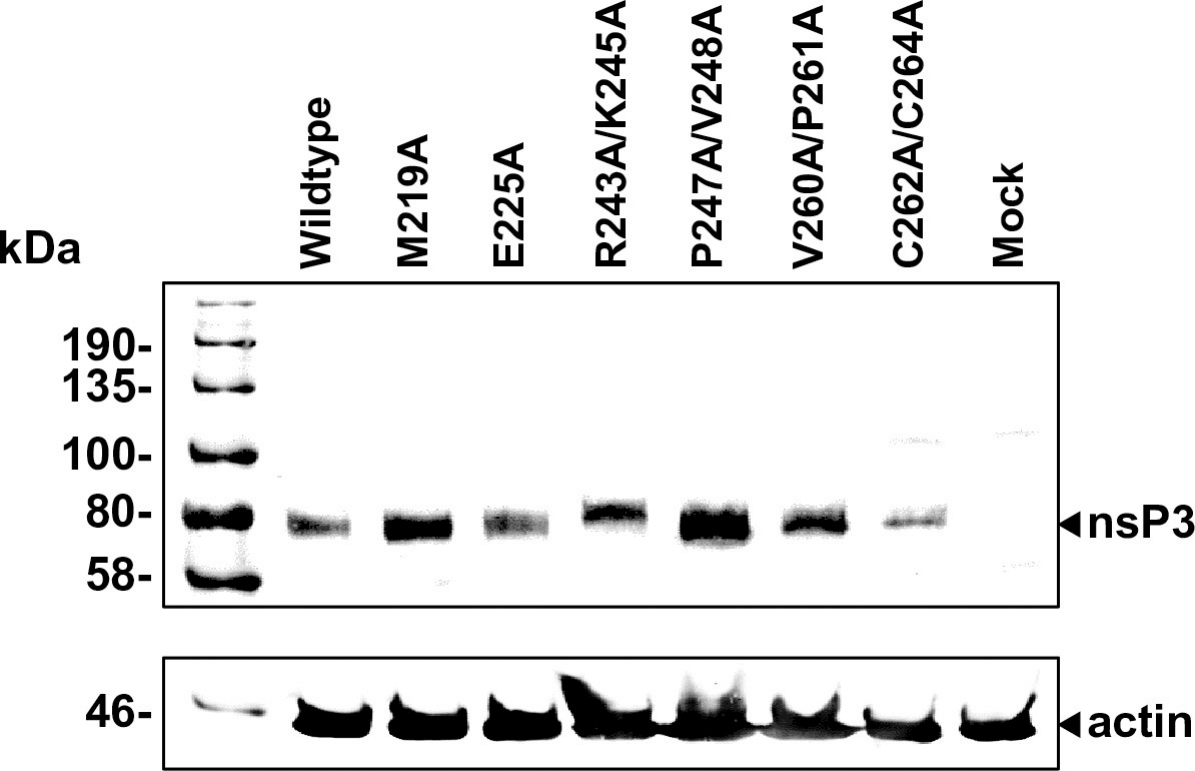
Expression of nsP3 in C2C12 cells. Cells were transfected with wildtype and the indicated mutant pcDNA3.1-nsP3 plasmids and cell lysates harvested at 48 h.p.t. into GLB. Lysates were analysed for expression of nsP3 by western blot. Actin was also detected by western blot as a loading control.

We wished to confirm that none of the phenotypes observed were due to destabilisation of the protein, however the lack of replication of some mutants meant that it was not possible to detect nsP3 by western blotting of replicon transfected cell lysates. To address this we therefore generated a panel of nsP3 mutants in pcDNA3, these plasmids were transfected into C2C12 cells and nsP3 expression detected by western blotting. As shown in Fig 5, all the mutants were expressed to a similar level as wildtype and did not exhibit any degradation patterns.

### Role of the AUD in the context of infection of mammalian cells with CHIKV

**Fig 6:**
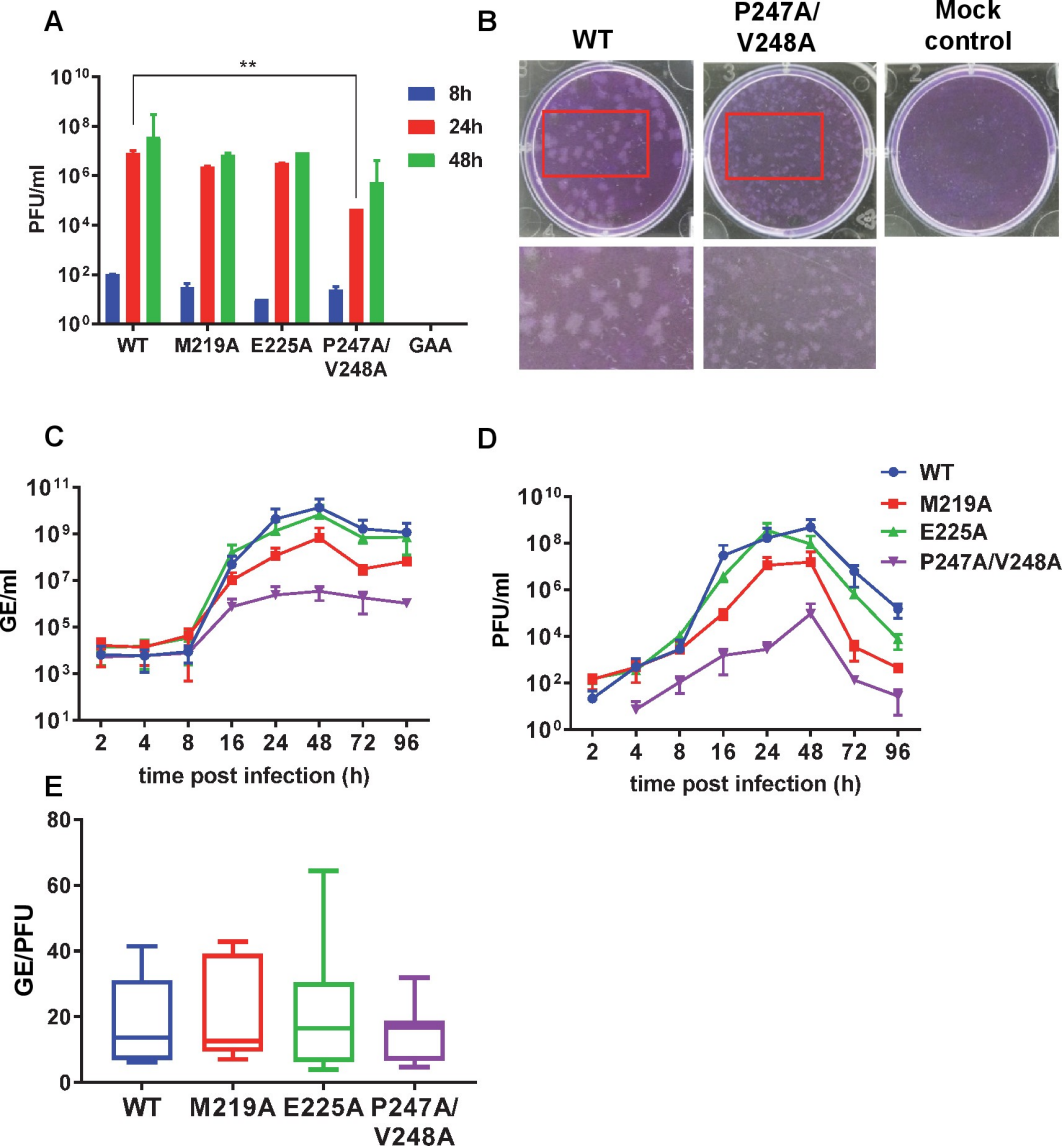
Phenotype of AUD mutations in the production of infectious virus. (A) ICRES-RNAs, wildtype (WT) and indicated mutants, were electroporated into C2C12 cells and supernatants were collected at 48 h.p.e. Virus was titrated by plaque assay in BHK-21 cells. Significant difference denoted by ** (P<0.01), compared to wildtype. (B) Plaques for wildtype and P247A/V248A were visualised illustrating the small plaque phenotype for this mutant. (C-E) C2C12 cells were infected with CHIKV (wildtype and indicated AUD mutants) at an MOI of 0.1. Supernatants were collected at the indicated times for genome RNA quantification (qRT-PCR) (C) and virus titration by plaque assay (D). Data in A, C and D are displayed as the means ± S.E. of three experimental replicates. (E) The ratios of genome RNA:infectivity were determined from (C) and (D) at 16, 24 and 48 h.p.i. and presented graphically.

We then sought to determine if the AUD played any role in other stages of the virus lifecycle. To test this, a subset of mutations that were able to replicate in all, or some of, the mammalian cells tested (M219A, E225A and P247A/V248A), together with the nsP4 GAA mutant as negative control, were introduced into an infectious CHIKV construct, derived from the same ECSA isolate as the replicons (LR2006 OPY1) [15,16]. Capped, *in vitro* transcribed virus RNA was electroporated into C2C12 cells and production of infectious virus was assessed by plaque assay of cell supernatants at 8, 24 and 48 h. post-electroporation (h.p.e.). C2C12 cells were chosen as our previous analysis had revealed that CHIKV grew to very high titres in these cells [17], and they are physiologically relevant, being muscle derived. As expected (Fig 6A), wildtype CHIKV produced a high titre of infectious virus following electroporation of C2C12 cells whereas the nsP4 GAA mutant did not produce any infectious virus. M219A and E225A were indistinguishable from wildtype, but P247A/V248A showed a significantly lower titre (approx. 10-fold reduced). We sought to confirm that the P247A/V248A phenotype was not directly attributable to the introduced mutations, and was not influenced by reversion or compensatory mutations elsewhere in the genome. To do this we generated RT-PCR amplicons spanning the entire coding sequence of the genome (primer sequences shown in S1 Table), using RNA derived from cells at 48 h.p.e. as template (termed P0). This analysis revealed that not only was the P247A/V248A mutation retained, but there was no evidence for any compensatory mutations (S4 Fig). Consistent with the data obtained for the R243A/K245A mutant replicon in C6/36 cells (Fig 4C), we observed that this mutant virus had already fully reverted to the wildtype sequence at 48 h.p.e. in C2C12 cells (S5 Fig), hence we discontinued work with this mutant. For both M219A and E225A no reversion was observed (S5 Fig) so we conclude that these two residues are not required for CHIKV replication.

During the course of these assays we noted that the P247A/V248A mutant uniquely exhibited a much smaller plaque size than the wildtype (Fig 6B). We reasoned that this might reflect a defect in either virus production or spread that could be masked in the electroporation procedure due to the high level of input RNA. To test this we performed a one-step growth assay by infecting C2C12 cells at an MOI of 0.1 with either wildtype CHIKV or the 3 mutants (M219A, E225A and P247A/V248A). Cell supernatants were harvested at various times post infection (h.p.i.) and analysed for genomic RNA by qRT-PCR (Fig 6C), and infectivity by plaque assay (Fig 6D). In order to exclude the possibility that the P247A/V248A mutant phenotype resulted in a delay to virus replication we extended this analysis to 96 h.p.i. Wildtype, M219A and E225A showed a rapid increase in both genomic RNA and infectivity between 8–48 h.p.i., reaching very high titres (for wildtype: 3.4x10^10^ RNA copies/ml and 4.7x10^8^ pfu/ml), and declining thereafter (likely due to cell death at later timepoints after infection). In contrast, levels of P247A/V248A accumulated very slowly, reaching a maximum of 4.6 x10^6^ RNA copies/ml and 2.8x10^5^ pfu/ml at 48 h.p.i. Levels of both RNA and infectious virus for P247A/V248A also declined after 48 h.p.i. confirming that the phenotype of this mutant was not associated with a delay to virus replication. Interestingly, direct comparison of the genomic RNA quantification with the infectivity over all timepoints revealed that the specific infectivity values for all four viruses were indistinguishable (Fig 6E). We conclude that, although P247A/V248A exhibited a defect in production of virus particles, the virions produced were equally infectious as wildtype.

**Fig 7:**
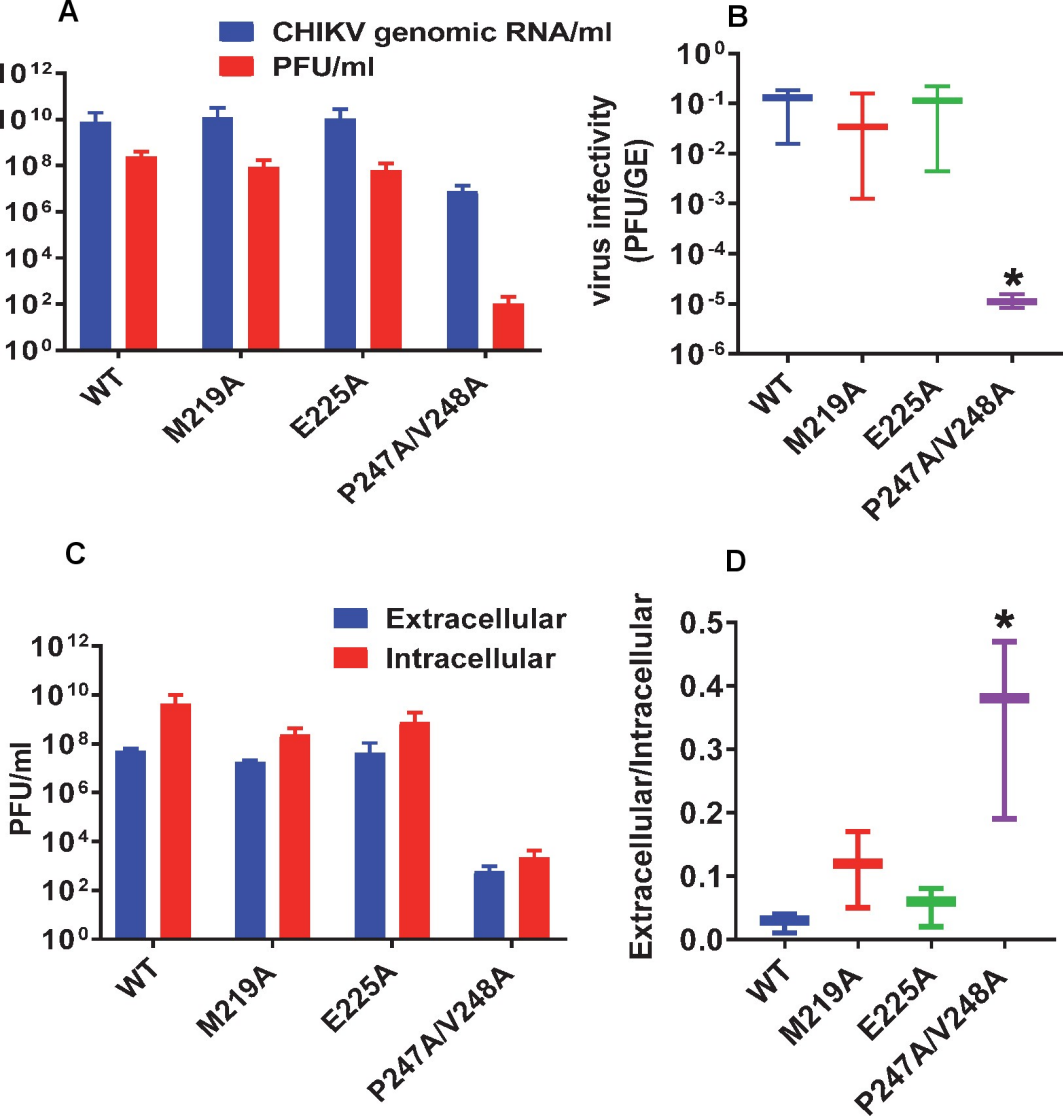
Phenotype of AUD mutations in virus entry, release and assembly. (A) C2C12 cells were infected with CHIKV (wildtype (WT) and indicated AUD mutants) at MOI of 1. At 24 h.p.i, cells were washed with PBS and resuspended in 1 ml fresh medium. Cell suspensions were freeze/thawed 3 times to release intracellular virus. Genome RNA was quantified by qRT-PCR (blue bars), and virus titrated by plaque assay (red bars). (B) Graphical representation of the ratio of infectivity to genomic RNA. (C) Extracellular (blue bars) and intracellular (red bars) viruses were collected at 36 h.p.e from C2C12 cells electroporated with the indicated ICRES RNAs, and titrated by plaque assay. (D) Graphical representation of the ratio of extracellular to intracellular virus titres. Significant difference denoted by * (P<0.05) compared to wildtype. Data in A and C are displayed as the means ± S.E. of three experimental replicates.

We then asked whether the reduced virus titre exhibited by P247A/V248A resulted from a defect in virus assembly or release from infected cells. To address this question we first analysed levels of viral genomic RNA (by qRT-PCR) and infectious virus (by plaque assay) present at 24 h.p.i. within cells infected with wildtype or the 3 mutants (at an MOI of 1). This analysis (Fig 7A) revealed that the levels of intracellular genomic RNA for wildtype, M219A and E225A were comparable whereas P247A/V248A showed a 1000-fold reduction. This was consistent with the replicon data–for P247A/V248A genomic RNA levels were reduced from a mean of 7.9 x 10^9^ RNA copies/ml to 7.4x10^6^. Levels of infectious virus were similar for wildtype, M219A and E225A, however, P247A/V248A showed a dramatic 10^7^-fold reduction in the amount of intracellular infectious virus compared to wildtype: from 2.4 x 10^8^ pfu/ml to 9.8 x 10^1^ pfu/ml. This was paralleled by a dramatic change in the ratio of genomic RNA:infectivity (Fig 7B), suggesting that although P247A/V248A exhibited a defect in genome replication there was an additional, more substantial phenotype in the production of infectious virus particles. The difference in magnitude of these two phenotypes suggests that they represented different functions of the AUD. To provide further support for this observation, we performed a similar experiment in which C2C12 cells were electroporated with wildtype or the 3 mutant virus RNAs (Fig 7C and 7D). This analysis revealed that the ratio of extra- to intracellular virus titres was significantly higher for P247A/V248A compared to wildtype and the other two mutants. We conclude that, although P247A/V248A produces less infectious virus, this can subsequently be released from the infected cells more efficiently than wildtype and the other two mutants.

### The P247A/V248A mutation selectively impairs subgenomic RNA synthesis

**Fig 8:**
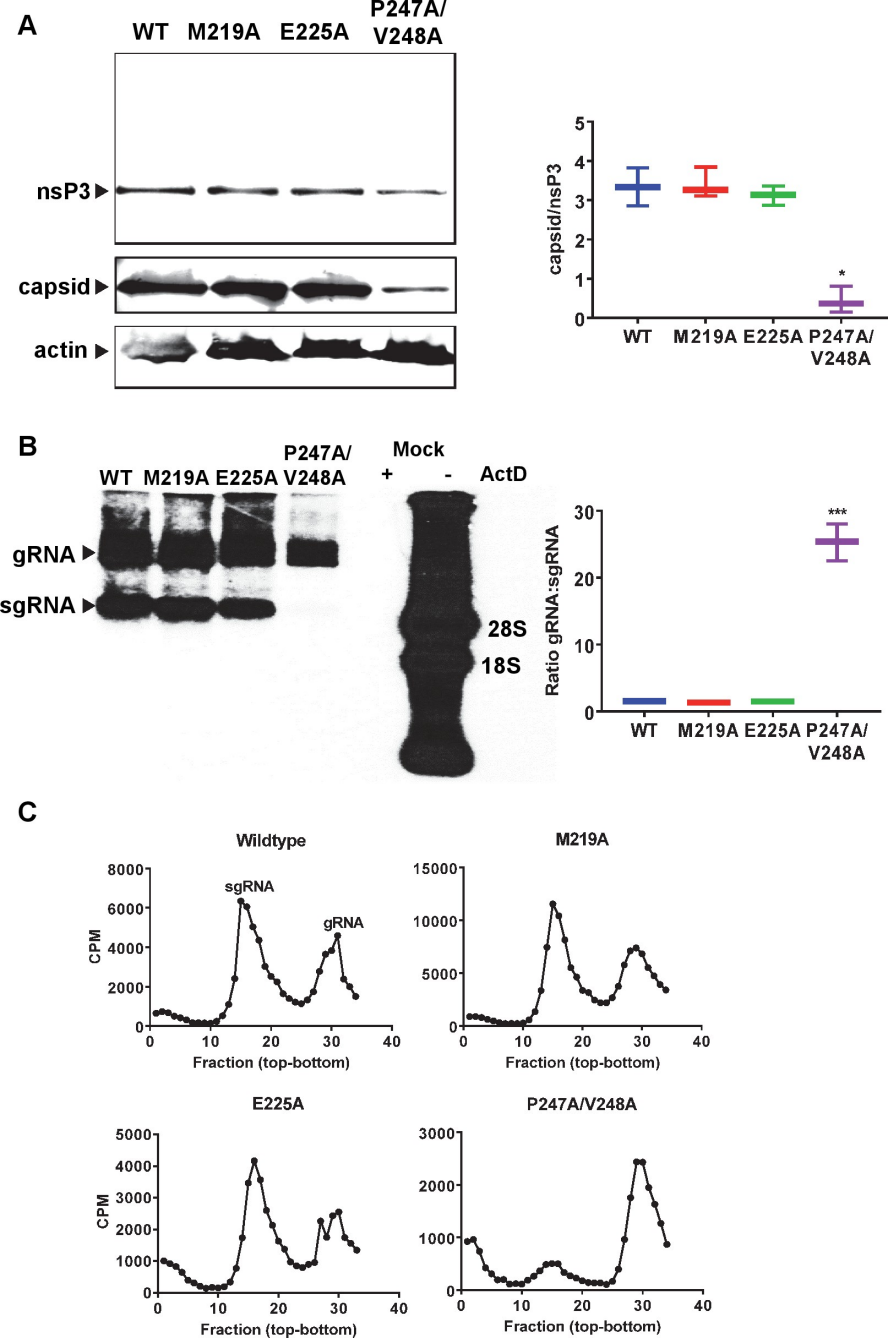
Effect of AUD mutations on CHIKV protein expression and RNA synthesis. (A) C2C12 cells were electroporated with ICRES-RNAs (wildtype (WT) and indicated AUD mutants) and cell lysates were collected at 36 h.p.e. Expression of nsP3 and capsid was analysed by western blot. Representative western blots are presented. For quantification using a LiCor Odyssey Sa fluorescence imager western blots from three independent experiments were analysed and the graph on the right shows the ratio of capsid to nsP3 expression. (B) C2C12 cells were electroporated with the indicated ICRES RNAs, cellular RNA synthesis was inhibited by actinomycin D and nascent viral RNAs were labelled with [^3^H]-uridine. A representative autoradiograph is presented. The graph on the right shows the ratio of gRNA to sgRNA determined by quantification of three independent experiments. (C) The same RNAs were fractionated on a sucrose gradient and [^3^H]-labelled RNAs were detected by scintillation counting of individual fractions.

We considered that the reason for the reduction in virus assembly exhibited by P247A/V248A could be due to a direct role of nsP3 in this process, or alternately could result from a defect in the production of the structural proteins. To test the second of these two possibilities, we analysed electroporated cells by western blot for the presence of both nsP3 and the capsid protein. P247A/V248A exhibited a modest reduction in nsP3 expression but a much greater reduction in the level of capsid expression compared to wildtype or the other two mutants. Indeed the ratio of capsid to nsP3 expression determined from the western blot analysis was approximately 10-fold lower for P247A/V248A (Fig 8A). During alphavirus replication the non-structural proteins (including nsP3) are translated from the full-length genomic RNA (gRNA), whereas the capsid and other structural proteins are translated from a subgenomic RNA (sgRNA). Transcription of both positive sense RNAs is mediated by a complex of the four nsPs using the full-length negative strand as template. This complex either initiates transcription from the 3′ end of the negative strand or from the subgenomic promoter. We hypothesised that the reduction in capsid expression for P247A/V248A could result from a defect in sgRNA transcription. To test this, C2C12 cells were electroporated and treated with actinomycin D (ActD) to block cellular RNA synthesis, prior to labelling with [^3^H]-uridine. Cellular RNA was extracted and analysed by MOPS-formaldehyde gel electrophoresis and autoradiography. As shown in Fig 8B, for wildtype, M219A and E225A, 2 radiolabelled species corresponding to gRNA and sgRNA were detected. However, for P247A/V248A, the sgRNA was present at very low levels, indeed almost undetectable. The corresponding ratio of gRNA:sgRNA for P247A/V248A (25.3:1) was significantly higher than that of wildtype (1.5:1). As controls, mock electroporated cells treated with ActD contained no [^3^H]-labelled RNA species, whereas in the absence of ActD the expected smear of [^3^H]-labelled RNAs with predominant bands corresponding to 18S and 28S ribosomal RNAs were observed (Fig 8B). To confirm these results, the harvested RNAs were also analysed by sucrose gradient centrifugation. Consistent with the electrophoretic analysis, wildtype, M219A and E225A showed two peaks corresponding to sgRNA and gRNA, whereas P247A/V248A exhibited a dramatically reduced sgRNA peak (Fig 8C).

### Effect of the P247A/V248A mutation on the RNA-binding activity of the AUD

**Fig 9:**
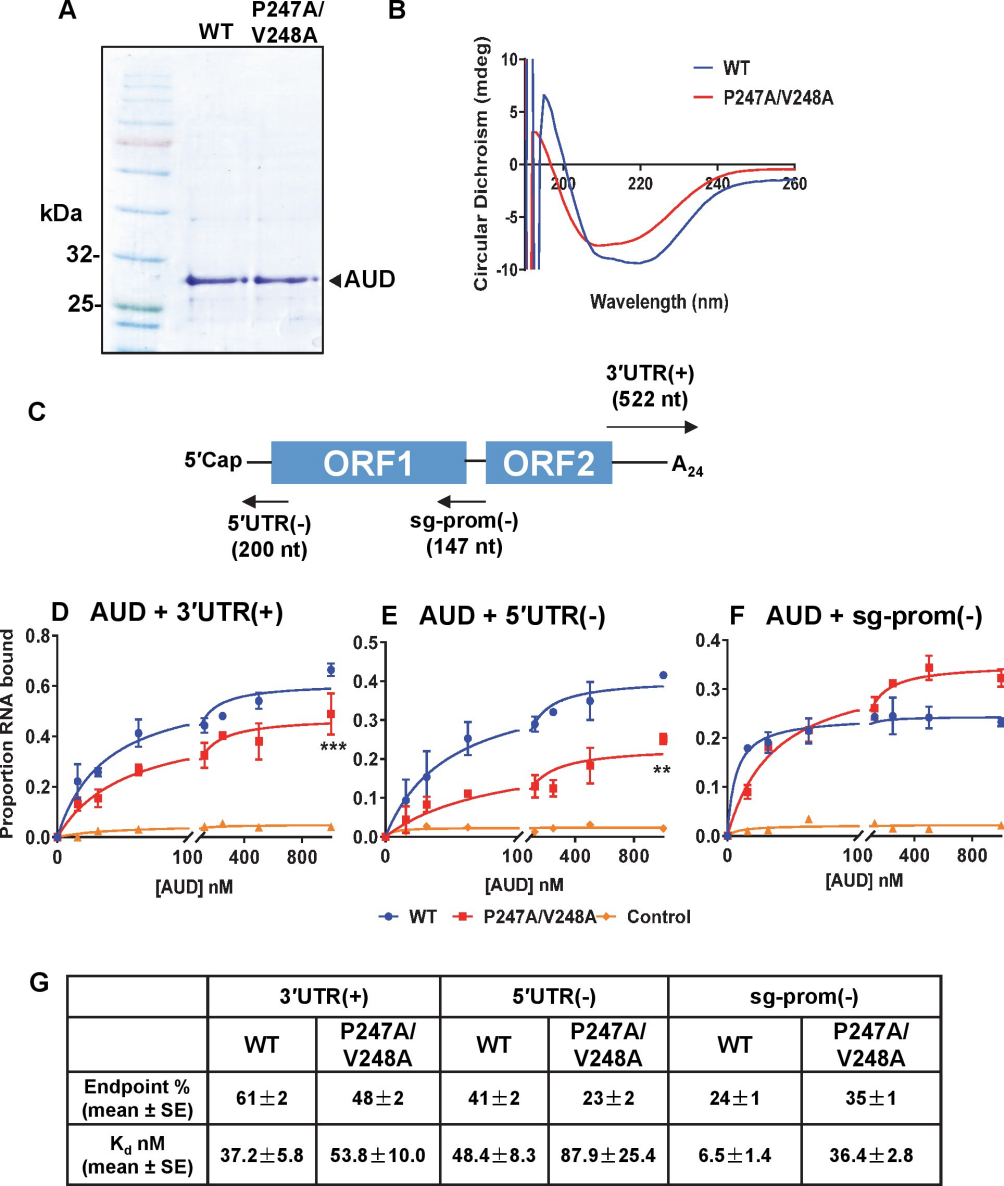
*In vitro* AUD RNA-binding activity. (A) *E*. *coli* expressed AUD (wildtype (WT) and P247A/V248A) were analysed by SDS-PAGE and Coomassie Brilliant blue staining. (B) Circular Dichroism analysis of purified AUD. (C) Schematic of the CHIKV genome showing the location of the various short RNA fragments used in subsequent filter binding analysis. (D-F) Filter binding analysis of the interaction between AUD and the indicated RNA fragments. Purified AUD at the indicated concentrations was incubated with radiolabelled RNA (1 nM) before application to a slot blot apparatus, filtering through nitrocellulose (protein-RNA complex) and Hybond-N (free RNA) membranes, and visualization by phosphoimaging. The negative control is wildtype AUD with an 80-mer aptamer raised against the foot-and-mouth disease virus 3D RNA-dependent RNA polymerase [18]. The percentage of RNA bound to the nitrocellulose membrane was quantified and plotted as a function of the AUD concentration. The data was fitted to a hyperbolic equation. Data are displayed as the means ± S.E. of three experimental replicates. (G) Endpoint (% of total RNA bound) and K_d_ values derived from the graphs in (D-F).

Our data thus far are consistent with the hypothesis that the P247A/V248A mutation results in a reduction in the ability of the nsP1-4 complex to recognise and initiate transcription from the subgenomic promoter. As the AUD has been predicted to possess RNA-binding activity [14], and initiation of gRNA or sgRNA transcription by the nsP1-4 complex will require specific recognition of cognate RNA sequences on the CHIKV negative strand, we further postulated that the phenotype of the P247A/V248A might be explained by a defect in RNA binding activity. To test this we expressed wildtype and P247A/V248A AUD mutants in *E*.*coli* as His-Sumo fusion proteins. The AUDs were cleaved from the fusion proteins by Sumo-protease and analysed by SDS-PAGE. As shown in Fig 9A, the AUDs could be purified to a high degree of homogeneity and exhibited the expected apparent molecular weights (~ 29 kDa). Circular dichroism (CD) analysis (Fig 9B) revealed that, as expected, the AUDs comprised predominantly α-helix with no significant differences in the overall structure as a result of the mutations. To test for RNA-binding activity we performed a filter binding assay [20] using purified AUDs and a radiolabelled RNA corresponding to the 3′ end of the CHIKV genome (3′UTR(+)), the 3′ end of the genomic negative strand RNA (5′UTR(-)), or the negative strand subgenomic promoter (sg-prom(-)) (Fig 9C). Both wildtype and P247A/V248A AUD were able to bind the 3′UTR(+) (Fig 9D) and 5′UTR(-) (Fig 9E), however P247A/V248A exhibited a significant increase in K_d_ values and decrease in maximal binding levels (endpoints) compared to wildtype. As 3′UTR(+) and 5′RNA(-) are involved in the initiation of negative and positive strand genome RNA synthesis, respectively; impaired binding of the P247A/V248A mutant AUD may explain the observed defect in CHIKV genome replication (Fig 3). For binding to the sg-prom(-) RNA (Fig 9F), P247A/V248A AUD showed a different phenotype with both a higher endpoint and K_d_ than wildtype. K_d_ and endpoint values are listed in Fig 9G. It is intriguing that at higher concentrations the P247A/V248A AUD bound to the sg-prom(-) RNA more efficiently than wildtype, this might possibly reflect some cooperativity in binding of AUD to this RNA sequence which could be enhanced by the mutation. However this is unlikely to be physiologically relevant as it only occurs at very high AUD concentrations. Taken together, these data suggest that the P247A/V248A defect in sgRNA synthesis may be in part explained by a reduction in the ability to specifically bind the subgenomic promoter.

**Fig 10:**
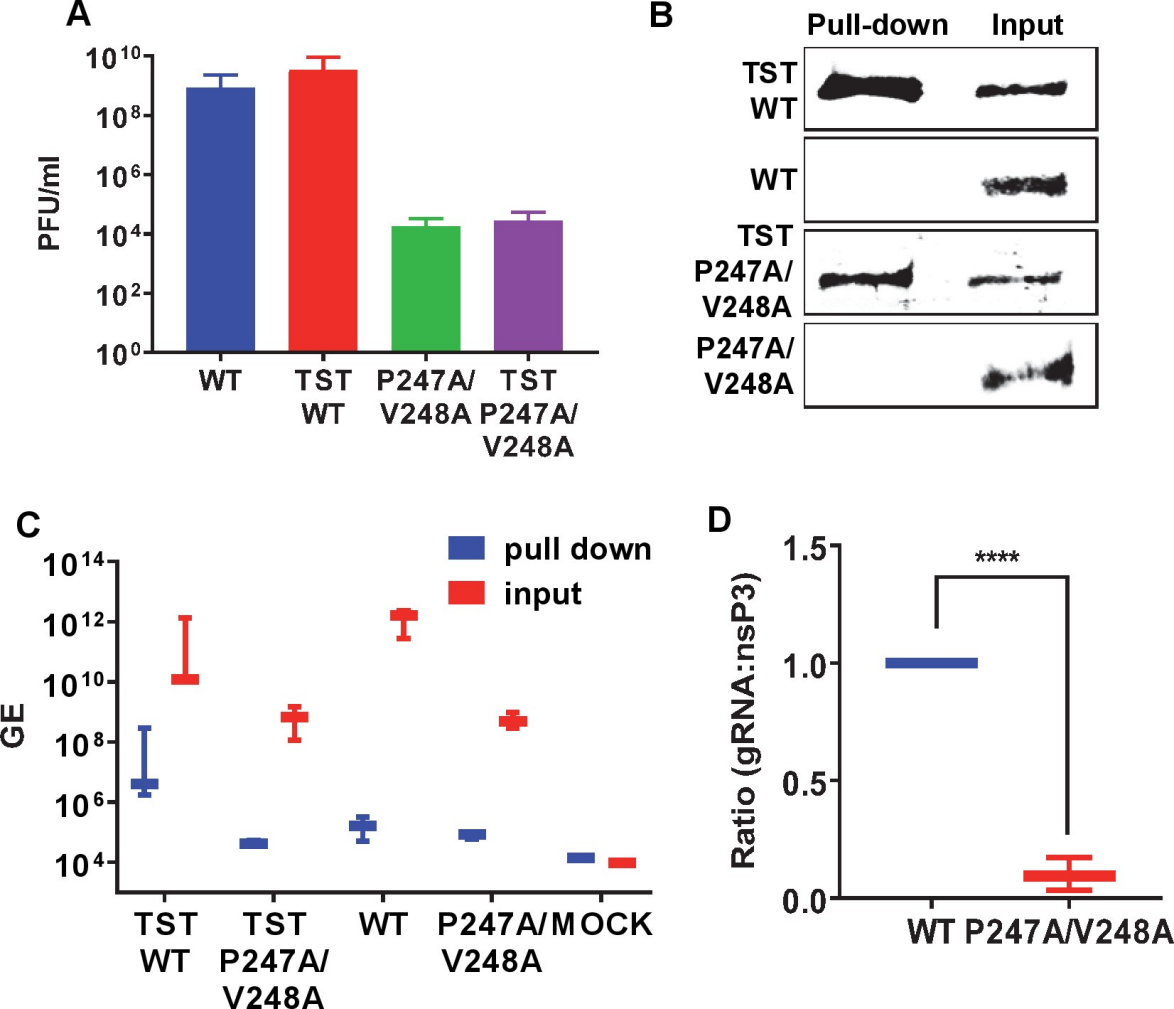
CHIKV genome RNA association with nsP3 during virus replication. C2C12 cells were electroporated with ICRES nsP3-TST or untagged ICRES RNAs (wildtype (WT) and P247A/V248A). (A) Supernatants were harvested at 48 h.p.e and titrated by plaque assay on BHK-21 cells. Data are displayed as the means ± S.E. of three experimental replicates. Cell lysates were collected at 60 h.p.e. and nsP3-TST was precipitated with Streptactin-sepharose beads. Bound proteins and input lysates were subjected to western blotting (B) and both co-precipitated RNAs and lysate samples were extracted by TRIzol and quantified by qRT-PCR (C). The ratio of gRNA to nsP3 is depicted graphically (D).

To explore the RNA binding activity of nsP3 to CHIKV genomic RNA during virus replication, we exploited a previously generated derivative of the ICRES infectious clone in which a twin-strep tag (TST) was introduced in frame near the C-terminus of nsP3, allowing efficient affinity purification of nsP3 by streptactin chromatography. We had previously used this experimental approach to investigate protein-protein and protein-RNA interactions of the hepatitis C virus NS5A protein [21–23]. We first confirmed that the presence of the TST did not adversely affect CHIKV virus replication: C2C12 cells were electroporated with either wildtype or P247A/V248A mutant CHIKV RNAs with either untagged or TST-tagged nsP3. Supernatants were harvested at 48 h.p.e and titrated by plaque assay on BHK21 cells. As shown in Fig 10A, the presence of the TST tag had no effect on the virus titre, but consistent with Fig 6 the titre of the P247A/V248A mutant viruses were significantly lower than wildtype. NsP3 proteins were purified from cell lysates on streptactin beads and both lysates and precipitated material analysed by western blot for nsP3 (Fig 10B) and qRT-PCR to determine the amount of gRNA associated with nsP3 (Fig 10C). Consistent with the *in vitro* RNA filter binding assay data, TST-P247A/V248A nsP3 bound at least 100-fold less gRNA compared to TST-wildtype nsP3 (Fig 10C), indeed the TST-P247A/V248A values were close to the limit of detection and comparable to mock (uninfected) values. To confirm these data we quantified the amount of nsP3 protein and gRNA from 3 separate experiments and determined the ratio. As shown in Fig 10D, when the gRNA:nsP3 ratio for wildtype was arbitrarily set to 1:1, the corresponding ratio for P247A/V248A was 0.095+/-0.06:1.

### Sub-cellular localisation of nsP3, capsid and dsRNA during CHIKV replication

**Fig 11:**
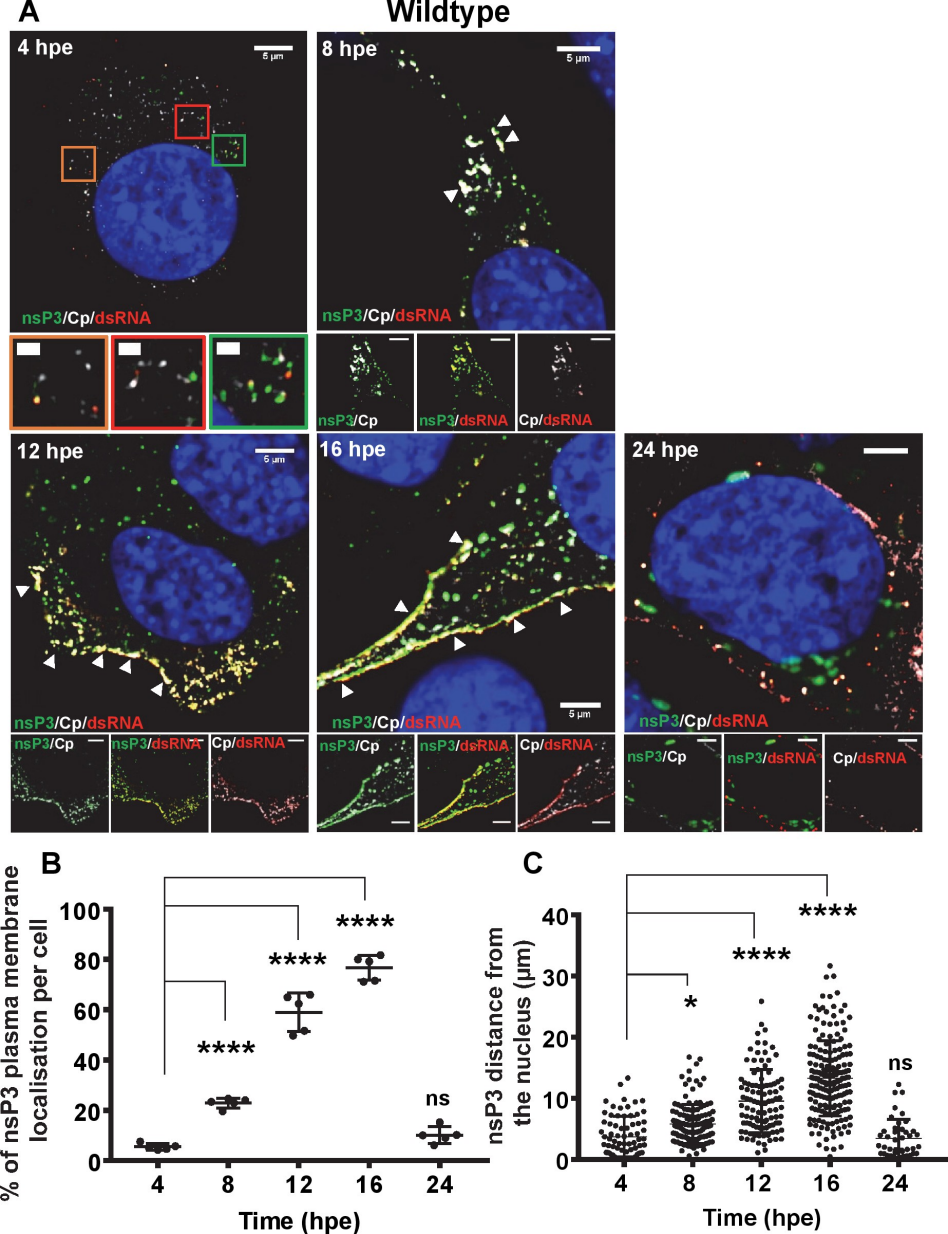
Fluorescence microscopy analysis of nsP3, capsid and dsRNA distribution during infection of C2C12 cells with wildtype CHIKV. C2C12 cells were electroporated with ICRES-nsP3-ZsGreen-CHIKV RNA. Cells were fixed at the indicated time points post-infection and stained with antibodies to capsid protein (Cp) (white) and dsRNA (red). (A) Green: nsP3-ZsGreen fusion, blue: nuclear DAPI counterstain. The scale bars are 5 μm and 1 μm, respectively. (B) The percentage of nsP3 that localised on the plasma membrane was determined for 5 cells. (C) The distance of individual nsP3 punctae from the nuclear membrane was determined for 5 cells. Localisations were calculated using Fiji. Significant differences denoted by * (P<0.05), or **** (P<0.0001), compared to the 4 h.p.e. results.

**Fig 12:**
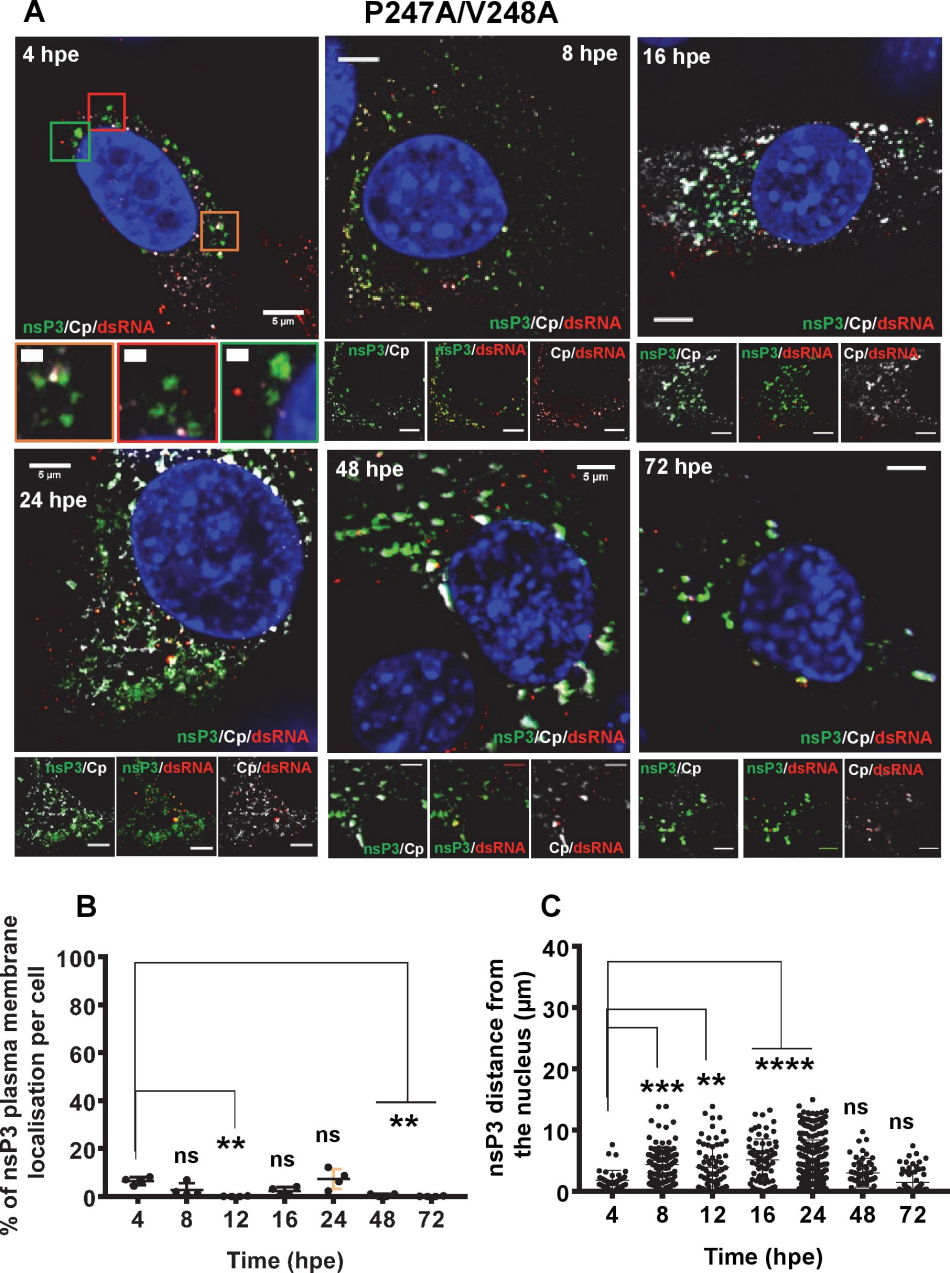
Fluorescence microscopy analysis of nsP3, capsid and dsRNA distribution during infection of C2C12 cells with P247A/V248A mutant CHIKV. C2C12 cells were electroporated with ICRES-nsP3-ZsGreen-CHIKV-P247A/V248A RNA. (A) Cells were fixed at the indicated time points post-infection and stained with antibodies to capsid protein (Cp) (white) and dsRNA (red). Green: nsP3-ZsGreen fusion, blue: nuclear DAPI counterstain. The scale bars are 5 μm and 1 μm, respectively. (B) The percentage of nsP3 that localised on the plasma membrane was determined for 5 cells. (C) The distance of individual nsP3 punctae from the nuclear membrane was determine for 5 cells. Localisations were calculated using Fiji. Significant differences denoted by ** (P<0.01), *** (P<0.001), or **** (P<0.0001), compared to the 4 h.p.e. results.

The effect of P247A/V248A on the nsP3:gRNA interaction suggested that this mutation might also disrupt the subcellular localisation of nsP3 in relation to both replication complexes and sites of virion assembly. To test this we exploited another derivative of the ICRES infectious CHIKV clone in which ZsGreen was inserted into nsP3 at the same position as the TST tag [15,24]. C2C12 cells were electroporated with ICRES-nsP3-ZsGreen-CHIKV RNAs (wildtype or P247A/V248A), and cells were analysed by confocal laser scanning microscopy (CLSM) with Airyscan for the distribution of nsP3, capsid (as a marker for virion assembly sites) and dsRNA (as a marker of genome replication) at different times post-electroporation. For wildtype at 4 h.p.e. (Fig 11A), small clusters of nsP3, capsid and dsRNA appeared in the cytoplasm but there was little co-localisation. By 8 h.p.e., nsP3, capsid and dsRNA co-localised in larger clusters, these appeared to accumulate at the plasma membrane at 12 and 16 h.p.e., by which time the majority of nsP3, capsid and dsRNA were co-localised on plasma membrane. By 24 h.p.e., it was clear that the infection cycle was complete as there was a reduction in levels of nsP3, capsid and dsRNA. Interestingly, capsid and dsRNA were still co-localised at the plasma membrane while most nsP3 was perinuclear. To confirm these observations we quantified both the amount of nsP3 that was plasma membrane localised (Fig 11B), and the distance of nsP3 from the nuclear membrane (Fig 11C), in 5 infected cells. Both of these parameters increased steadily until 16 h.p.e., and declined sharply thereafter. In contrast, P247A/V248A exhibited a very different distribution pattern of all three markers throughout the infection cycle (Fig 12). Consistent with the western blot data (Fig 8A) levels of capsid were lower than wildtype at all timepoints, but in addition the co-localisation of nsP3, capsid and dsRNA at the plasma membrane was not observed. This was confirmed by quantitative analysis of both plasma membrane localisation (Fig 12B), and distance from the nucleus (Fig 12C) of nsP3. We confirmed that this was not due to a delay in virus replication as no plasma membrane localisation was observed even at 72 h.p.e., this is consistent with the time-course of virus production shown in Fig 6C and 6D. We conclude that, as well as disrupting sgRNA transcription and production of the structural proteins, the P247A/V248A mutant phenotype also manifests in a perturbation to the sub-cellular localisation of nsP3. The loss of plasma membrane localisation may contribute to the reduced level of infectious virus production.

## Discussion

Of the four alphavirus non-structural proteins, nsP3 remains the least well understood [25]. The protein consists of three domains, the N-terminal of which has been identified as a macrodomain that binds to ADP-ribose and possesses ADP-ribosylhydrolase activity [9]. Recent studies have proposed a role for this enzymatic activity in virus pathogenesis but as yet the underlying mechanisms remain elusive [11]. The C-terminal hypervariable domain differs dramatically in amino acid sequence between different alphaviruses and is intrinsically disordered. It has been shown to interact with a range of cellular proteins, including components of stress granules [26], and is implicated in the assembly of virus genome replication complexes.

In contrast, we know virtually nothing about the function of the central AUD domain. The fact that this domain is highly conserved between different alphaviruses suggests that it plays a fundamental role in the virus lifecycle. Detailed structural information about the AUD is available however, as the partial structure of the SINV nsP2-nsP3 precursor, including the C-terminal protease and methyltransferase-like domains of nsP2 and the macro and AUD domains of nsP3, has been determined [14]. This analysis revealed that the AUD contained an unique zinc-binding fold with four cysteine residues coordinating a zinc molecule, this formed part of a putative RNA binding surface. Mutagenesis of two of these cysteines revealed an essential role in virus replication. Our data agree with this observation, as the C262A/C264A mutant failed to replicate in any cell type tested.

Mutation of two residues adjacent to the zinc-binding cysteines, V260A/P261A, also completely abrogated CHIKV genome replication. Although adjacent in the primary amino acid sequence, these residues are located on the distal face of the AUD (Fig 1B), suggesting that they are not involved in zinc binding, but may instead interact with key cellular factor(s) or play an alternative structural role.

In contrast, the other mutants generated during this study exhibited a number of distinct cell-type and species-specific phenotypes (summarised in Table 1). Mutation of two surface exposed basic residues (R243 and K245) abrogated replication in all mammalian cells but showed full replication capability in mosquito cells. However, this apparent discrepancy could be explained by the observation that these two mutations rapidly reverted to wildtype in mosquito cells but failed to do so in C2C12 cells (Fig 4C). These data indicate that R243 and K245 are required for CHIKV genome replication. We do not have an explanation for why R243A/K245A, but not the other lethal mutations, was able to revert in mosquito cells. However it is noteworthy that the reversion to the wildtype sequence took 72 h in C6/36 cells, suggesting that perhaps the lack of cytopathology of CHIKV replication in mosquito cells could facilitate the replication of a minority species. Interestingly, the sequence trace at 24 h in C6/36 shows the presence of such a minority species that would encode a Thr at 243 and 245, suggesting that the two basic residues are not absolutely required.

**Table 1:** AUD mutant replication phenotypes in different cell types.

CHIKV-D-*luc* SGR	Human			Rodent		Mosquito	
	Huh7	Huh7.5	RD	C2C12	BHK	U4.4	C6/36
**Wildtype**							
**M219A**							
**E225A**							
**R243A/K245A**							
**P247A/V248A**							
**V260A/P261A**							
**C262A/C264A**							

**Key:** ■ Wildtype replication, ■ impaired replication, ■ no replication. ■ reversion

M219 was also of particular interest as mutation of this residue had no significant effect on genome replication in any cell type apart from U4.4 mosquito cells. M219A replicated well in C6/36 mosquito cells and the key difference between these two cell lines is that C6/36 have a defect in the RNAi response due to a Dcr2 mutation [19]. We propose therefore that M219 may be involved in the interaction of nsP3 with a component of the mosquito RNAi response to inhibit this key mosquito antiviral pathway. We are currently undertaking proteomic and functional analysis to test this hypothesis.

In the second part of this study we focused on P247A/V248A to address the molecular mechanism underpinning the phenotype of this mutant. In the context of the subgenomic replicon P247A/V248A showed a variety of phenotypes from complete lack of replication in Huh7 and Huh7.5 cells (Fig 2), to a 10-fold reduction in other mammalian and mosquito cells (Fig 3). It is intriguing that the P247A/V248A mutant exhibited a cell-type and species-specific phenotype as this implies that a cell factor may contribute to the phenotype. For example, a cell factor that is not expressed in Huh7 cells might be involved in the function of nsP3 in transcribing the subgenomic RNA. An alternative explanation is that Huh7 and Huh7.5 cells support lower levels of CHIKV replication than all of the other cell types in this study. The fold increase in FLuc values between 4–12 h.p.e. was 36-fold (Huh7) and 94-fold (Huh7.5) (Fig 2). This compared to 150-fold (RD), 740-fold (C2C12), 250-fold (BHK), 177-fold (U4.4) and 310-fold (C6/36). These overall higher levels of replication may have resulted in the P247A/V248A values rising above the threshold for detection. Virus data in C2C12 cells were consistent with replicon results: following electroporation of viral RNA P247A/V248A showed a modest but significant defect in virus infectivity and exhibited a small plaque phenotype. Further study indicated that P247A/V248A was competent in virus entry and virus release, however, it exhibited a major defect in assembly of infectious virus particles. This defect led to both a delay and a reduction in the release of infectious virus, consistent with the small plaque phenotype. The molecular mechanism underpinning the P247A/V248A defect was shown to be a reduction in subgenomic RNA synthesis, leading to a concomitant reduction in the expression of the structural proteins. It is noteworthy that when analysed in the context of the replicon in C2C12 cells, the P247A/V248A mutant exhibited a 10-fold reduction in FLuc, but RLuc was higher than wildtype. This is also consistent with a defect in transcription of the subgenomic mRNA. The reduced affinity of P247A/V248A AUD for the CHIKV sg-prom(-) RNA as demonstrated by the RNA filter binding assay may help to explain this, but it is possible that other effects of P247A/V248A, such as aberrant host protein recruitment, may also help to explain the defect in subgenomic RNA synthesis. Of note, P247A/V248A AUD also exhibited impaired binding to the 3′UTR(+) and 5′RNA(-) *in vitro*, and also bound less genomic RNA *in vivo*, compared to wildtype, consistent with an overall defect in RNA replication. However, it is important to note that whilst there was a difference between wildtype and P247A/V248A in the ratio of genomic RNA to nsP3 in TST precipitates (Fig 10D), this may have resulted, at least in part, from the reduced abundance of genomic RNA in the P247A/V248A infected cells. Previous studies have also shown that nsP3 is important for initial replication complex formation and negative strand RNA synthesis [27]. Taken together we propose that the AUD plays a critical role in all stages of CHIKV RNA synthesis, but particularly in the transcription of the sgRNA.

Analysis of the distribution of nsP3, capsid and dsRNA during CHIKV replication by confocal microscopy revealed further insights into the P247A/V248A phenotype. Wildtype nsP3 exhibited a high level of co-localisation with dsRNA at all time points up to 16 h.p.e. (Fig 11A), consistent with the role of nsP3 in genome replication. At 12 and 16 h.p.e., both nsP3 and dsRNA also co-localised with capsid and were concentrated at the plasma membrane. In contrast, P247A/V248A nsP3 showed not only a reduction in the co-localisation with dsRNA, but also a loss of the plasma membrane accumulation (Fig 12A). A simple explanation for this observation is that capsid accumulates at the plasma membrane and recruits both nsP3 and dsRNA, and this was perturbed by the low level of capsid expression exhibited by the P247A/V248A mutant. However we cannot rule out the possibility that there is a role for nsP3 (and the AUD in particular) in coordinating the processes of genome replication and virus assembly to facilitate production of infectious virus particles at the plasma membrane. This is in agreement with early evidence for a juxtaposition of sites of genome replication, viral protein translation and nucleocapsid assembly in the case of Sindbis virus [28]. In addition it may be that nsP3 has a role in the trafficking of nucleocapsids from these sites (cytoplasmic vacuoles (CPVs)) to the plasma membrane.

In conclusion we propose that the nsP3 AUD is a multi-functional domain. It is not only a critical determinant of both cell and species-specificity, but also plays roles in virus genome replication and assembly. The rational mutagenesis of the CHIKV AUD described here is the first detailed structure-function analysis of this domain and raises many questions. In particular, we need to determine what cellular and viral proteins are interaction partners for the AUD, and investigate how these interactions shed light on the phenotypes of AUD mutants? We expect that our current proteomic analysis, exploiting both a novel SNAP-tagged nsP3 recently developed in our laboratory [29] and a twin-strep tag (TST) approach which we recently used to identify interacting partners of the hepatitis C virus NS5A protein [21], will provide some of the answers to these questions. We also hope that these studies will help to identify targets for antiviral intervention and means of rational attenuation for vaccine development.

## Materials and methods

### Sequence alignment

AUD amino acid sequences from a number of different alphaviruses (Sindbis, Ockelbo, O’Nyong-Nyong, Ross River, Semliki Forest, Fort Morgan, Venezuelan, Eastern and Western Equine Encephalitis, Highlands-J and CHIKV) were obtained from NCBI and aligned by Clustal Omega. The predicted locations of conserved residues were then identified by Pymol, taking the Sindbis nsP2/3 protein structure (PDB ID code 4GUA) [14] as a reference.

### Cell culture

Mammalian cells Huh7, Huh7.5, RD, C2C12 and BHK-21 were maintained at 37°C with 5% CO_2_ in DMEM supplemented with 10% FCS, 0.5 mM non-essential amino acids and penicillin-streptomycin (100 units/mL). Huh7 cells were obtained from John McLauchlan (Centre for Virus Research, Glasgow), Huh7.5 cells from Charles Rice (Rockefeller University, New York), RD cells from Nicola Stonehouse (University of Leeds), C2C12 cells from Michelle Peckham (University of Leeds) and BHK-21 cells from John Barr (University of Leeds). *Aedes albopictus* mosquito cell lines (U4.4 and C6/36) were obtained from Susan Jacobs (The Pirbright Institute) and maintained in Leibovitz's L-15 media supplemented with 10% FBS, 10% tryptose phosphate broth and penicillin-streptomycin (100 units/mL). Mosquito cells were incubated at 28°C without CO_2_.

### Plasmid constructions

A fragment including the AUD coding sequence was excised from the CHIKV-D-Luc-SGR plasmid, and inserted into pcDNA3.1 to generate pcDNA3.1-AUD. This was used as a template for site-directed (Quikchange) mutagenesis using specific primers (primer sequences available upon request) to produce the required AUD mutations. Finally, the AUD mutated fragments were excised and religated into the CHIKV-D-Luc-SGR. AUD fragments were subsequently excised from CHIKV-D-Luc-SGRs and ligated into ICRES-CHIKV-WT. For the twin-strep tag (TST) derivatives of wildtype and P247A/V248A, synthetic oligonucleotides (sequence available upon request) were used to substitute the appropriate coding sequence [21] for the RLuc in nsP3. For expression of nsP3 alone in eukaryotic cells the entire nsP3 coding sequences (flanked by an appropriate Kozak consensus/AUG at the 5′ end, and termination codon at the 3′ end) were amplified by PCR and cloned into the pcDNA3.1 vector. The nsP4 GAA mutant was generated by site-directed mutagenesis in the CHIKV-D-Luc-SGR, sequenced and recloned back into either ICRES-CHIKV-WT or CHIKV-D-Luc-SGR.

### Transfection and Dual-luciferase Assay

Capped RNAs were generated from the CHIKV-D-Luc-SGR for transfection using the mMACHINE SP6 transcription kit (ThermoFisher Scientific) and purified with PureLink RNA Mini Kit (Life Technologies). Routinely 0.5 μg CHIKV-D-Luc-SGR was transfected into 10^6^ cells using Lipofectamine 2000 (Life Technologies) according to the manufacturer’s instructions. At 4, 12, 24 and 48 h post transfection (h.p.t.), cells were harvested and both Renilla and Firefly luciferase activity measured using the Dual-luciferase Assay System (Promega) according to the manufacturer’s instructions. Each sample had three repeats and the data shown in this study represent the mean of three experimental replicates (n = 3).

### Sequence analysis of subgenomic or viral RNA

Cytoplasmic RNA from electroporated or infected cells were Trizol extracted, prior to reverse transcription with random primers using SuperScript IV Reverse Transcriptase (Invitrogen) according to the manufacturer’s instructions. cDNAs were then used as a template to amplify fragments of the CHIKV genome with specific primers (primer sequences available upon request). PCR products were subjected to sequencing analysis.

### Titration of infectious CHIKV by plaque assay

ICRES-CHIKV RNAs were produced and purified as described above. C2C12 cells were electroporated with ICRES-CHIKV RNAs (1 μg RNA was electroporated into 1.2 x10^6^ cells) and incubated at 37°C. Cell supernatants were collected at 8, 24 and 48 hours post electroporation (h.p.e.), diluted with cell medium and applied to monolayers of BHK-21 cells for 1 h at 37°C. The inoculum was aspirated and plates were overlaid with 0.8% methylcellulose for 48 h at 37°C for plaque formation. For titration of intracellular viruses, cells were freeze/thawed 3 times and supernatants were collected by centrifugation at 12000×g for 10 min., prior to application to BHK-21 cells. All virus work was performed in a Biological Safety Level 3 (BSL3) laboratory. Plaques were visualised by photography with a Canon EOS 80D.

### One-step virus growth curve

Infectious CHIKV was harvested from C2C12 cells electroporated with ICRES RNAs at 48 h.p.e. Cell supernatants were titrated by plaque assay in BHK-21 cells and stored at -80°C. For virus growth kinetic analysis (one-step growth curve), C2C12 cells were infected with wildtype or AUD mutant CHIKV at an MOI of 0.1 for 1 h. Infected cells were washed three times with PBS and incubated with fresh complete medium at 37°C. For RNA quantification, RNAs were extracted from supernatants with Trizol (ThermoFisher Scientific). qRT-PCR for CHIKV genome RNA was performed with One-step MESA GREEN qRT-PCR MasterMix Plus for SYBR assay (Eurogentec) following the manufacturer’s instructions. Primers for qRT-PCR were designed to amplify a short sequence of nsP3: (nsP3-F: GCGCGTAAGTCCAAGGGAAT, nsP3-R: AGCATCCAGGTCTGACGGG). Genomic RNA equivalents were determined by reference to a standard curve. Plaque assay was performed as described above.

### CHIKV RNA synthesis

C2C12 cells were electroporated with ICRES RNAs. At 10 h.p.e. actinomycin D (1 μg/ml) was added and the cells were incubated for a further 2 h. [^3^H]-uridine (20 μCi/ml) was then added and the cells were incubated for an additional 3 h., at which time the monolayers were washed 3 times with ice-cold PBS, lysed and RNA extracted with TRIzol reagent.

For measurement of viral RNA synthesis, the harvested RNAs were separated on a MOPS-Formaldehyde gel. The gel was fixed (15% methanol, 10% acetic acid and 75% dH_2_O) for 30 min followed by fluorography (Fluorographic reagent Amplify, GE Healthcare) for another 30 min. Gels were dried for 2 h. before exposure to autoradiographic film at -80°C for 4 days.

For gradient analysis, equal volumes of harvested RNAs were loaded on to 14 ml 5–25% (w/v) sucrose gradients in 100 mM sodium acetate and 0.1% SDS followed by centrifugation at 150,000×g for 5 h. at room temperature. Gradients were fractionated into 350 μl fractions, and radioactivity of each fraction was determined by liquid scintillation counting.

### Analysis of CHIKV protein expression

C2C12 cells were electroporated with ICRES RNAs and incubated for 36 h. Cells were washed 3 times with PBS, lysed by resuspension in Glasgow lysis buffer (GLB) [1% Triton X-100, 120 mM KCl, 30 mM NaCl, 5 mM MgCl_2_, 10% glycerol (v/v), and 10 mM piperazine-N,N'-bis (2-ethanesulfonic acid) (PIPES)-NaOH, pH 7.2] supplemented with protease inhibitors and phosphatase inhibitors (Roche Diagnostics), and incubated on ice for 15 min. Following separation by SDS-PAGE, proteins were transferred to a polyvinylidene fluoride (PVDF) membrane and blocked in 50% (v/v) Odyssey blocking buffer (LiCor) in Tris-buffered saline (TBS) [50 mM Tris, 150 mM NaCl, pH 7.4]. The membrane was incubated with primary antibody in 25% (v/v) Odyssey blocking buffer overnight at 4°C, then incubated with fluorescently labelled anti-rabbit (800 nm) secondary antibodies for 1 h at room temperature (RT) before imaging on a LiCor Odyssey Sa fluorescence imager.

### Expression and purification of AUD proteins

The wildtype and P247A/V248A AUD (nsP3 residues Ile141 to Gly374) were cloned into pET-28a-His-sumo for expression in *Escherichia coli* and subsequent analysis. His-sumo tagged AUD expression plasmids were transformed into Rosetta 2 and cultures were grown in Luria-Bertani (LB) medium supplemented with 50 μg/μl ampicillin and 1% (w/v) glucose. The cells were grown at 37°C to an optical density at 600 nm (OD_600_) of 0.5 to 0.7 and then induced with IPTG (isopropyl-D-thiogalactopyranoside) (0.5 mM) for 5 h at 18°C. The cells were harvested by centrifugation at 7,000 rpm for 10 min. Wildtype or mutant AUD protein was purified by sequential His-tag affinity purifications. Briefly, cell pellets were suspended in 20 ml AUD lysis buffer (100 mM Tris-HCl pH 7, 200 mM NaCl, 20 mM imidazole) supplemented with 2 μg/μl DNase and EDTA-free protease inhibitor cocktail tablets (Roche). The cell suspension was lysed by sonication on ice at an amplitude of 10 μm for six pulses of 20 s separated by 20 s and the extract clarified by centrifugation at 16000×g for 30 min at 4°C. The supernatant was filtered through a 0.45 μm filter and applied to a Ni^2+^ His-tag column for purification. Purified proteins were dialyzed to remove imidazole and sumo-protease was added to cleave the His-sumo tag. After dialysis, the proteins were applied again to the His-tag purification column, and the flow-throughs were collected as purified AUD proteins.

### Circular dichroism (CD) spectroscopy

Far-UV CD spectroscopy was performed on an APP Chirascan CD spectropolarimeter to obtain the secondary structure of AUDs. Spectra (190–260 nm) were recorded using 200 μl protein solution (at a concentration of 0.2 mg/ml) in a 1 mm path-length cuvette. Protein CD spectra deconvolution was analysed by DichroWeb.

### RNA filter binding assay

To produce radiolabelled RNA transcripts the corresponding DNA sequences (Fig 9C) were PCR-amplified and cloned into pcDNA3.1. Plasmids were linearised and used as templates for *in vitro* transcription with T7 RNA polymerase, 500 μM each of ATP, CTP and GTP, 12 μM UTP, and 50 μCi [^32^P]-UTP for 2 h. After DNase treatment for 30 min., radiolabelled transcripts were purified with an RNA purification kit (Fisher Scientific). Radiolabelled transcripts and AUD proteins were diluted in binding buffer (40 mM Tris-HCl pH 7.5, 5 mM MgCl_2_, 10 mM DTT, 50 μg/ml bovine serum albumin, 10 μg/ml yeast tRNA [Ambion]) and pre-incubated separately for 10 min at 4°C. The binding reaction was initiated by mixing 1 nM radio-labelled RNA and AUD proteins (0 to 1 μM) in a 200 μl final volume at 4°C for 30 min. Membranes were pre-soaked in binding buffer supplemented with 5% (v/v) glycerol and assembled from bottom to top as follows in a slot-blot apparatus (Bio-Rad): filter paper, Hybond-N nylon (Amersham Biosciences) to bind free RNA molecules, and nitrocellulose (Schleicher & Schuell) to trap soluble protein-RNA complexes. After assembly, 200 μl of each binding reaction mixture was applied to each slot and filtered through the membranes. Each slot was washed with 0.5 ml of binding buffer and air dried, and quantification of radioactivity was performed using an image plate, BAS 1000 Bioimager (Fuji), and Aida Image Analyser v4.22 software. Fitting was performed using GraphPad Prism 5 software. In each case, the data were fitted to the hyperbolic equation R = R_max_ x R/(K_d_ + [P]), where R is the percentage of bound RNA, R_max_ is the maximal percentage of RNA competent for binding, [P] is the concentration of AUD, and K_d_ is the apparent dissociation constant.

### Precipitation of nsP3 and viral RNA

Co-precipitation experiments were performed in C2C12 cells electroporated with ICRES One-Strep-tag (OST) RNAs using Streptactin-agarose (Thermo Fisher Scientific), following the manufacturers protocol. Precipitated proteins were subjected to immunoblotting and co-precipitated RNAs were extracted by TRIzol and quantified by qRT-PCR.

### Fluorescence microscopy analysis of CHIKV-infected cells

The P247A/V248A mutation was introduced into ICRES-nsP3-ZsGreen-CHIKV where ZsGreen was fused into the hypervariable domain of nsP3. C2C12 cells were electroporated and harvested, fixed with 4% paraformaldehyde (PFA) at defined h.p.e., permeabilised by treatment with methanol, blocked with 2% BSA, and incubated with capsid protein antibody (gift from Andres Merits) or dsRNA antibody (J2 antibody, Scicons) at 4°C overnight, followed by secondary antibodies (Alexa Fluor 633 conjugated chicken anti-rabbit IgG and Alexa Fluor 594 conjugated donkey anti-mouse IgG) for 1h at room temperature. Distribution of nsP3, capsid protein and dsRNA were detected using a Zeiss LSM880 with Airyscan. Post-acquisition analysis was conducted using Zen software (Zen version 2015 black edition 2.3, Zeiss) or Fiji (v1.49) software [30].

### Statistical analysis

Statistical analysis was performed using unpaired two-tailed Student's t tests, unequal variance to determine statistically significant differences from the results for the wild type (n≥3). Data in bar graphs are displayed as the means ± S.E. of three experimental replicates (n = 3).

## Supporting information

S1 Fig A. Alignment of AUD amino acid sequences (nsP3 residues 210–276) of multiple alphaviruses indicating key residues mutated in this study. B. Ribbon structure of Sindbis virus AUD also showing location of mutated residues (PDB ID code 4GUA) [14]. Image constructed using PyMol.
(PPTX)

S2 Fig Absolute values for Firefly luciferase from replicon assays. The indicated cells were transfected with CHIKV-D-luc-SGR wildtype (WT) and mutant RNAs and harvested for Firefly luciferase assay at the indicated time points. Data normalised to the 4 h.p.t. timepoints are shown in Figs 2–4 in manuscript.
(PPTX)

S3 Fig Absolute values for Renilla luciferase from replicon assays. The indicated cells were transfected with CHIKV-D-luc-SGR wildtype (WT) and mutant RNAs and harvested for Renilla luciferase assay at the indicated time points. Data normalised to the 4 h.p.t. timepoints are shown in Figs 2–4 in manuscript.
(PPTX)

S4 Fig The P247A/V248A mutation did not revert or result in compensatory mutations elsewhere in the genome. ICRES-P247A/V248A RNA was electroporated into C2C12 cells and cytoplasmic RNA was TRIzol extracted at 48 h.p.e. cDNA was generated from the extracted cell RNA with random primers before PCR was performed with specific primers (see supplementary S1 Table). A. PCR fragments used for CHIKV whole genome sequencing. B. Sequencing alignment result between wildtype and P247A/V248A mutant using DNA Dynamo software. Red underlined sequences show changes from P247 (CCG) and V248 (GTG) to alanine (GCGGCG)
(PPTX)

S5 Fig Sequencing analysis of virus passage P0. P0: supernatant virus stock obtained from C2C12 cells at 48 h.p.e. nsP3 coding sequence was amplified by RT-PCR and sequenced. The region spanning the indicated mutations is shown. Note that for both E225A and R243A/K245A the sequence traces shown are from the negative strand, hence the colour of the trace does not match the colour code of the sequence below. R243A/K245A had already reverted to wildtype, whereas the other mutants had not reverted.
(PPTX)

S1 Table Primers used to amplify cDNA and sequence the ICRES-P247A/V248A virus for complementary mutations. (PPTX)
