# Supplementary figures and images for: Multiple roles of the non-structural protein 3 (nsP3) alphavirus unique domain (AUD) during Chikungunya virus genome replication and transcription
Source: PLoS Pathog. 2019 Jan 22;15(1):e1007239. doi: 10.1371/journal.ppat.1007239 (PMC6358111; doi:10.1371/journal.ppat.1007239)

## Slide 1
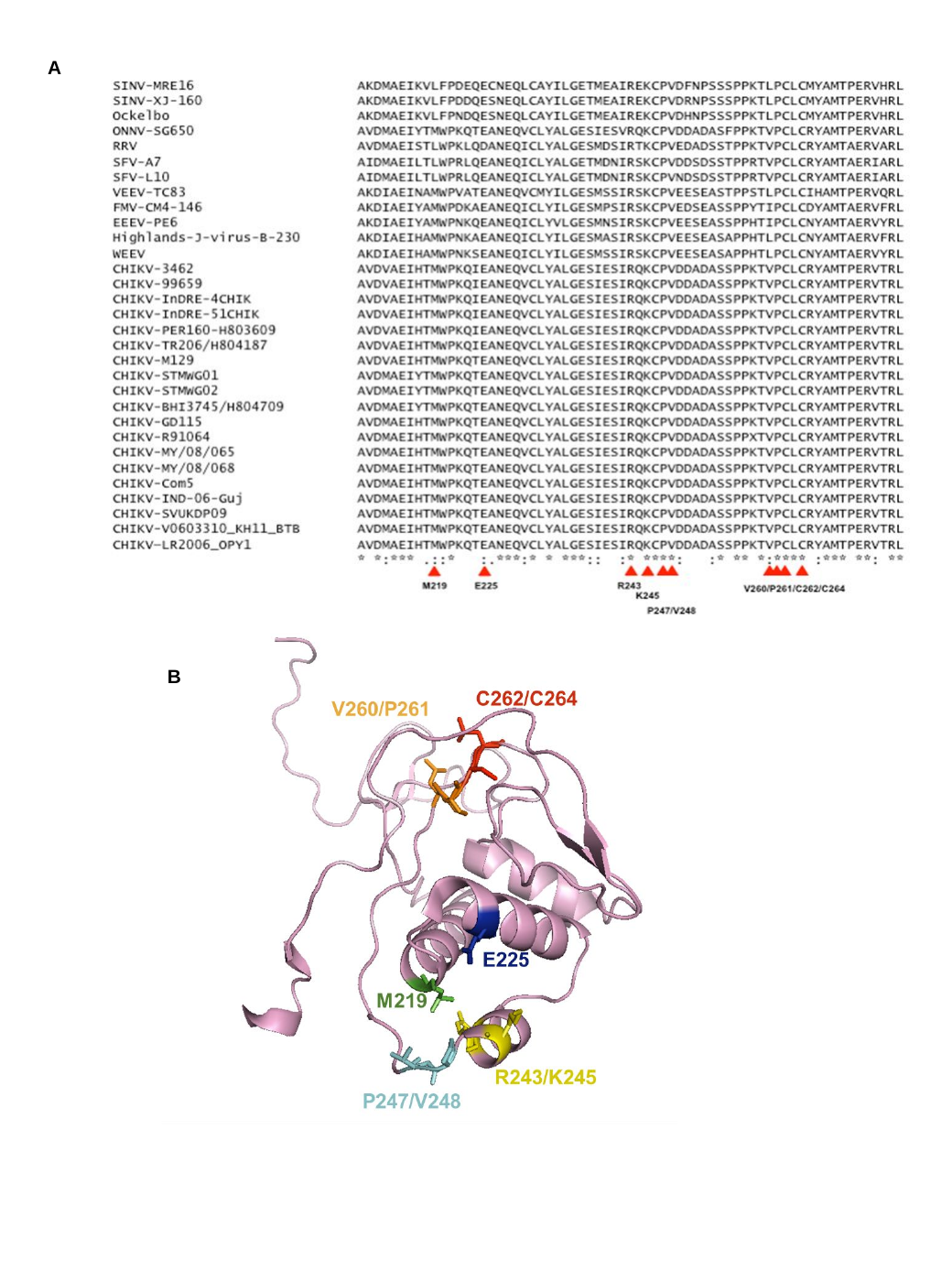

A
B

Supplement: S1 Fig — A. Alignment of AUD amino acid sequences (nsP3 residues 210–276) of multiple alphaviruses indicating key residues mutated in this study. B. Ribbon structure of Sindbis virus AUD also showing location of mutated residues (PDB ID code 4GUA) [14]. Image constructed using PyMol. (PPTX) [file ppat.1007239.s001.pptx]

## Slide 1
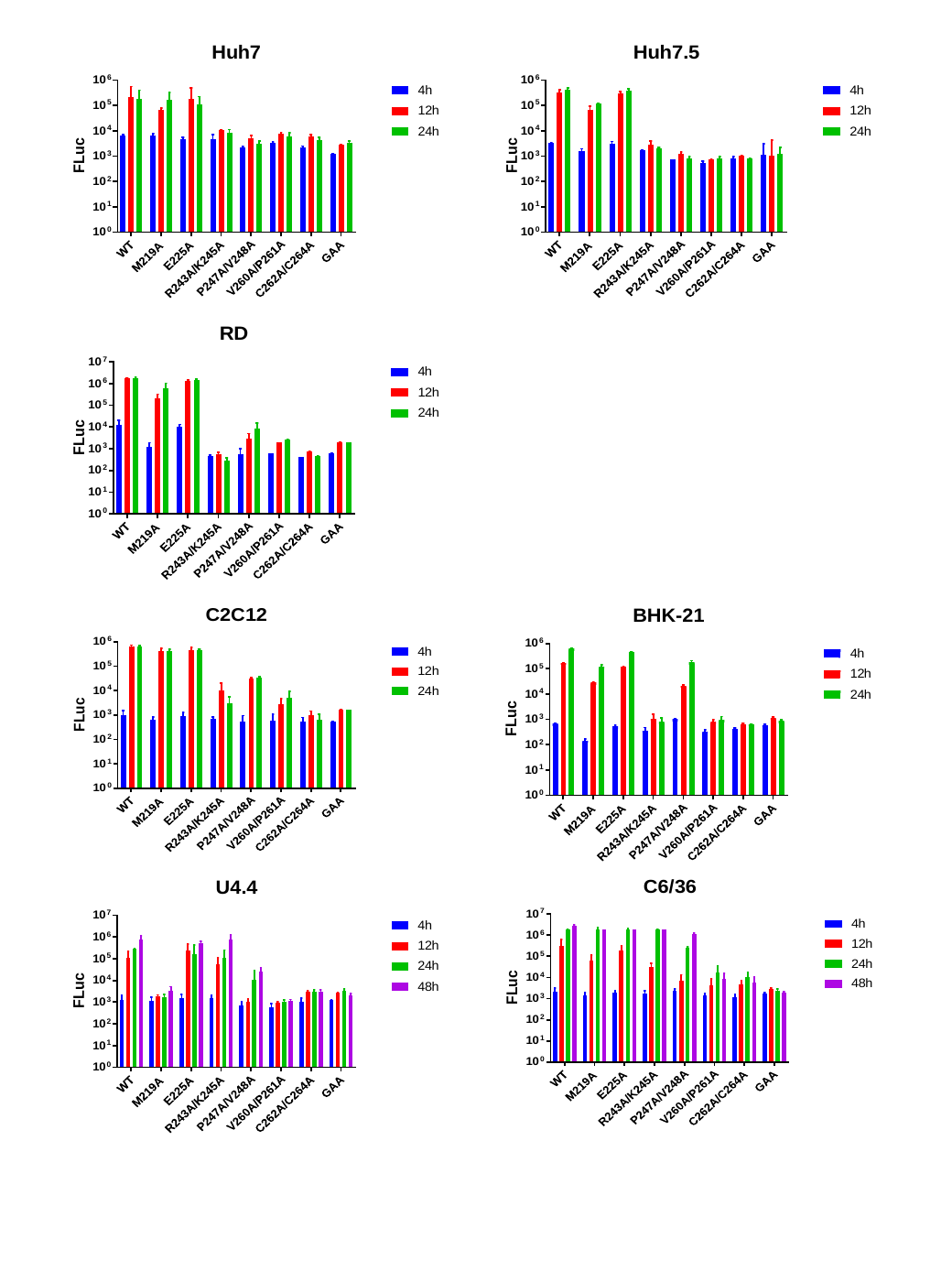

Supplement: S2 Fig — The indicated cells were transfected with CHIKV-D-luc-SGR wildtype (WT) and mutant RNAs and harvested for Firefly luciferase assay at the indicated time points. Data normalised to the 4 h.p.t. timepoints are shown in Figs 2–4 in manuscript. (PPTX) [file ppat.1007239.s002.pptx]

## Slide 1
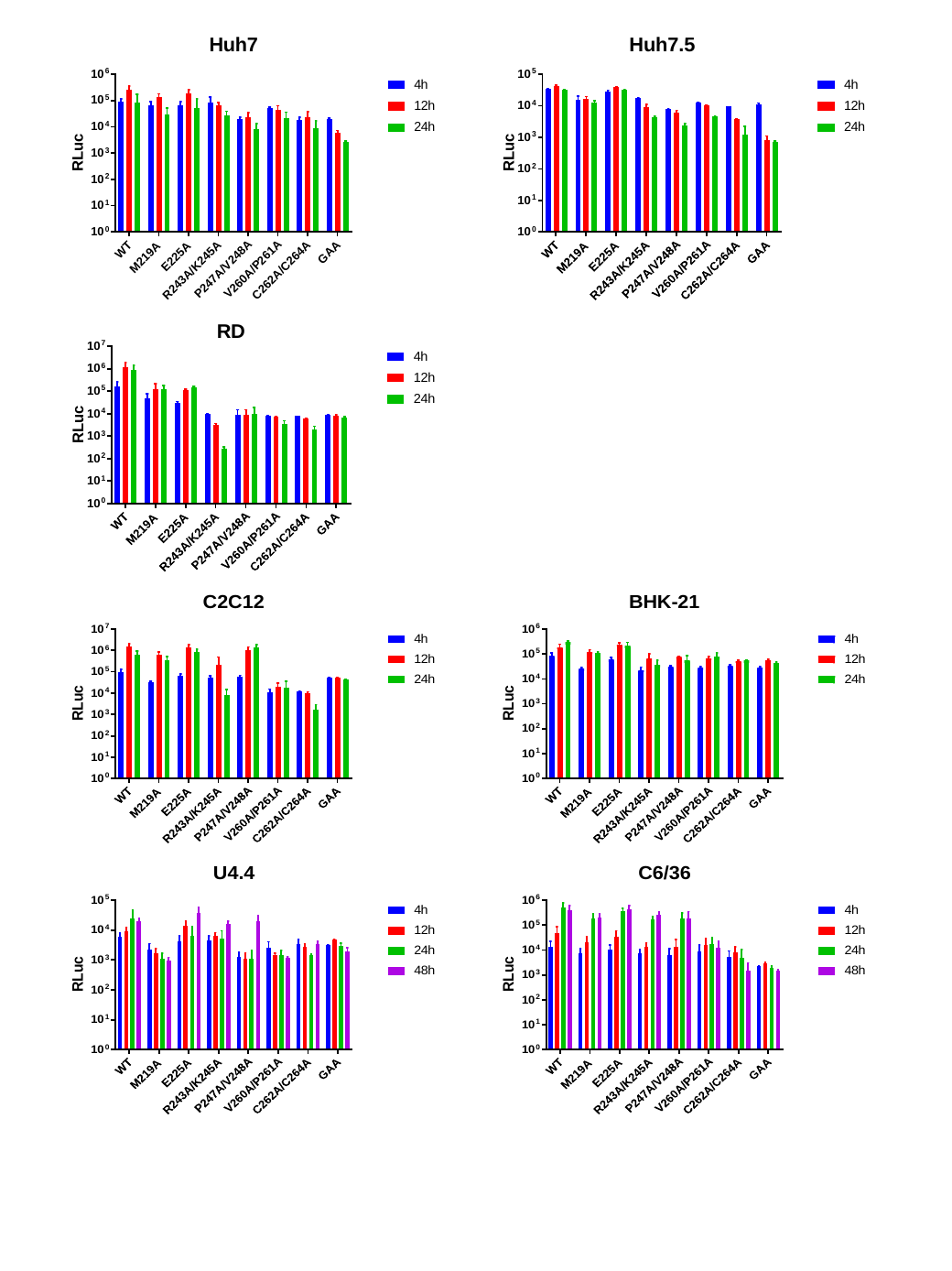

Supplement: S3 Fig — The indicated cells were transfected with CHIKV-D-luc-SGR wildtype (WT) and mutant RNAs and harvested for Renilla luciferase assay at the indicated time points. Data normalised to the 4 h.p.t. timepoints are shown in Figs 2–4 in manuscript. (PPTX) [file ppat.1007239.s003.pptx]

## Slide 1
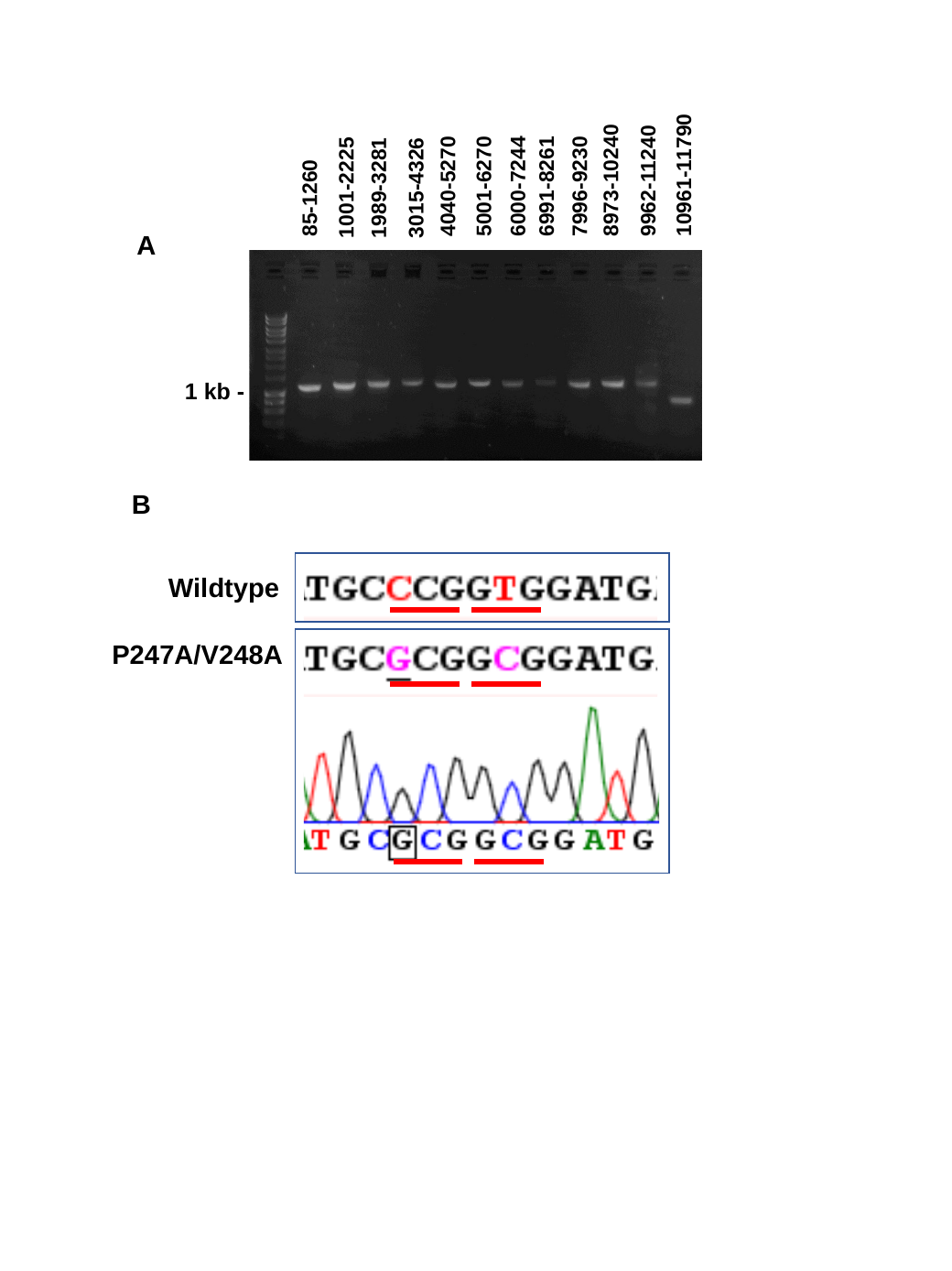

10961-11790
1989-3281
4040-5270
5001-6270
6000-7244
6991-8261
7996-9230
8973-10240
9962-11240
85-1260
1001-2225
3015-4326
A
1 kb -
B
Wildtype
P247A/V248A

Supplement: S4 Fig — ICRES-P247A/V248A RNA was electroporated into C2C12 cells and cytoplasmic RNA was TRIzol extracted at 48 h.p.e. cDNA was generated from the extracted cell RNA with random primers before PCR was performed with specific primers (see supplementary S1 Table). A. PCR fragments used for CHIKV whole genome sequencing. B. Sequencing alignment result between wildtype and P247A/V248A mutant using DNA Dynamo software. Red underlined sequences show changes from P247 (CCG) and V248 (GTG) to alanine (GCGGCG) (PPTX) [file ppat.1007239.s004.pptx]

## Slide 1
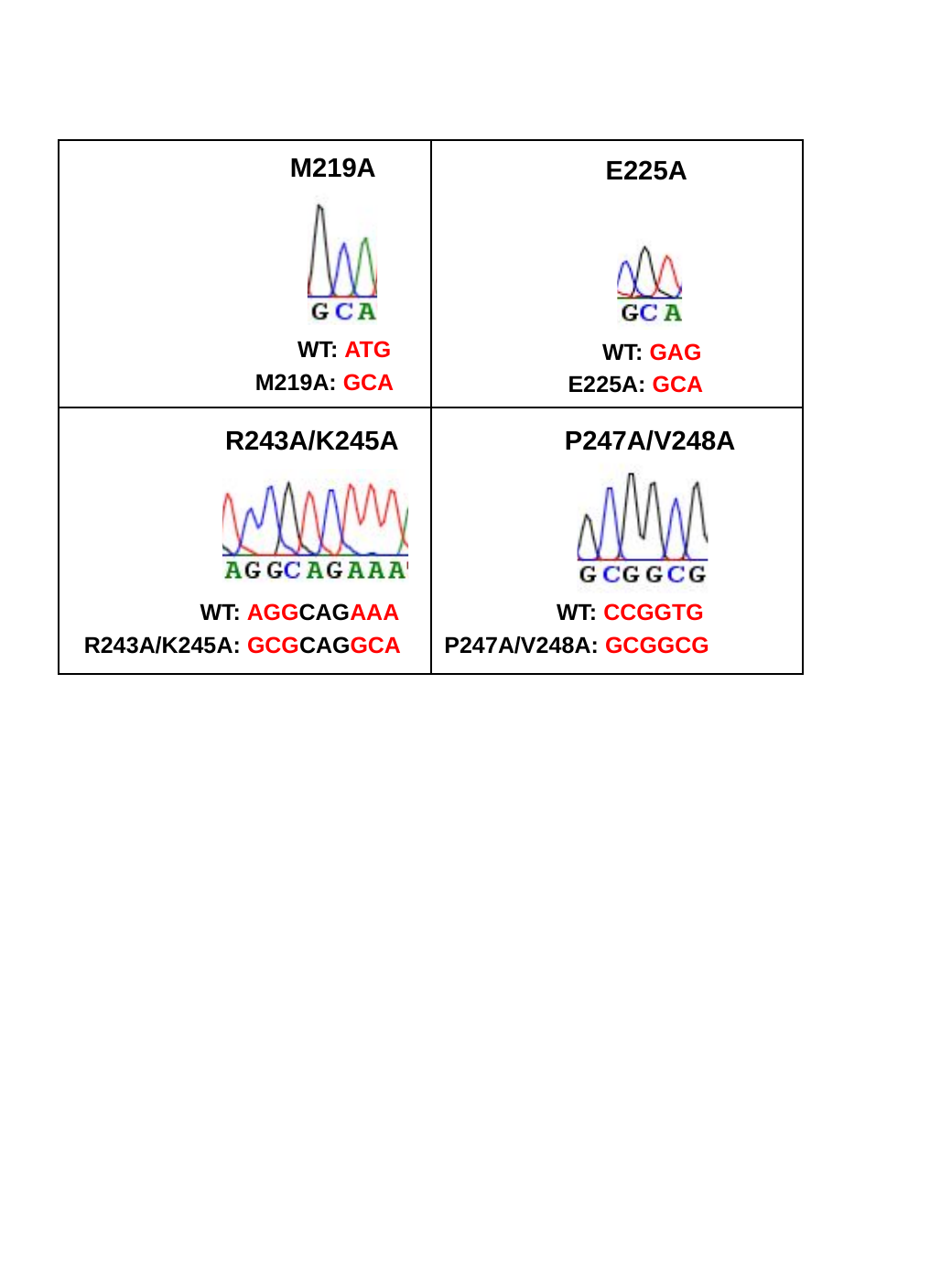

M219A
E225A
WT: ATG
WT: GAG
M219A: GCA
E225A: GCA
R243A/K245A
P247A/V248A
WT: AGGCAGAAA
WT: CCGGTG
P247A/V248A: GCGGCG
R243A/K245A: GCGCAGGCA

Supplement: S5 Fig — P0: supernatant virus stock obtained from C2C12 cells at 48 h.p.e. nsP3 coding sequence was amplified by RT-PCR and sequenced. The region spanning the indicated mutations is shown. Note that for both E225A and R243A/K245A the sequence traces shown are from the negative strand, hence the colour of the trace does not match the colour code of the sequence below. R243A/K245A had already reverted to wildtype, whereas the other mutants had not reverted. (PPTX) [file ppat.1007239.s005.pptx]
