# Supplementary material for: Multiple roles of the non-structural protein 3 (nsP3) alphavirus unique domain (AUD) during Chikungunya virus genome replication and transcription
Source: PLoS Pathog. 2019 Jan 22;15(1):e1007239. doi: 10.1371/journal.ppat.1007239 (PMC6358111; doi:10.1371/journal.ppat.1007239)
Supplement: S1 Table — (PPTX) [file ppat.1007239.s006.pptx]

## Slide 1
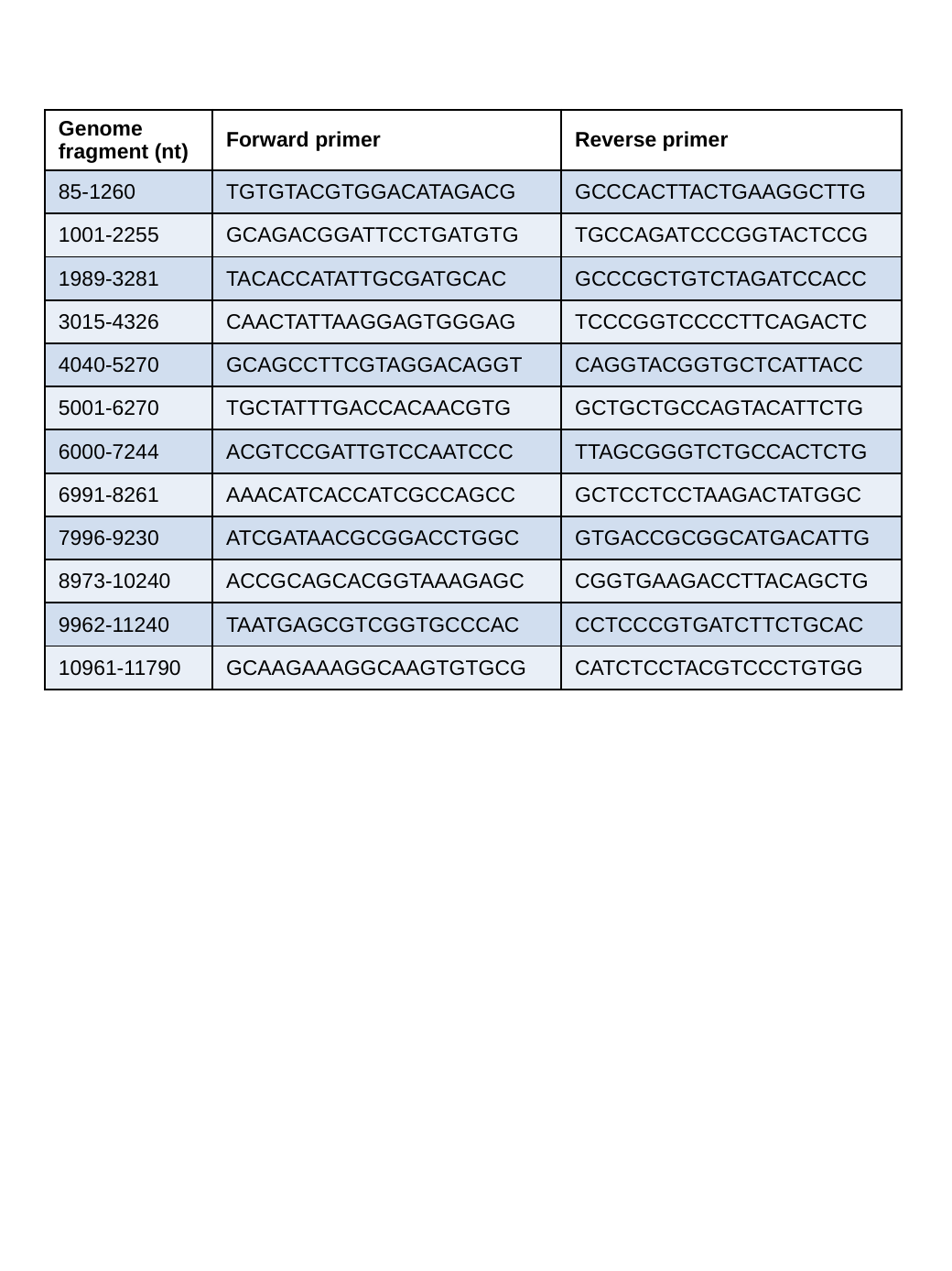

| Genome fragment (nt) | Forward primer | Reverse primer |
| --- | --- | --- |
| 85-1260 | TGTGTACGTGGACATAGACG | GCCCACTTACTGAAGGCTTG |
| 1001-2255 | GCAGACGGATTCCTGATGTG | TGCCAGATCCCGGTACTCCG |
| 1989-3281 | TACACCATATTGCGATGCAC | GCCCGCTGTCTAGATCCACC |
| 3015-4326 | CAACTATTAAGGAGTGGGAG | TCCCGGTCCCCTTCAGACTC |
| 4040-5270 | GCAGCCTTCGTAGGACAGGT | CAGGTACGGTGCTCATTACC |
| 5001-6270 | TGCTATTTGACCACAACGTG | GCTGCTGCCAGTACATTCTG |
| 6000-7244 | ACGTCCGATTGTCCAATCCC | TTAGCGGGTCTGCCACTCTG |
| 6991-8261 | AAACATCACCATCGCCAGCC | GCTCCTCCTAAGACTATGGC |
| 7996-9230 | ATCGATAACGCGGACCTGGC | GTGACCGCGGCATGACATTG |
| 8973-10240 | ACCGCAGCACGGTAAAGAGC | CGGTGAAGACCTTACAGCTG |
| 9962-11240 | TAATGAGCGTCGGTGCCCAC | CCTCCCGTGATCTTCTGCAC |
| 10961-11790 | GCAAGAAAGGCAAGTGTGCG | CATCTCCTACGTCCCTGTGG |
